# Coexisting Volatile and Nonvolatile Switching in 3D ALD‐IGZO Vertical RRAM for Fully Hardware‐Based Wide Reservoir Computing

**DOI:** 10.1002/advs.77076

**Published:** 2026-08-11

**Authors:** Seeun Lee, Jaewon Park, Nawoon Kim, Seohyeon Ju, Seong‐Cheol Jang, Hyun‐Suk Kim, Sungjun Kim

**Affiliations:** ^1^ Division of Electronics and Electrical Engineering Dongguk University Seoul Republic of Korea; ^2^ Department of Energy and Materials Engineering Dongguk University Seoul Republic of Korea

**Keywords:** ALD‐IGZO, in‐memory computing, neuromorphic device, vertical resistive random‐access memory, wide reservoir computing

## Abstract

Although indium‐gallium‐zinc oxide (IGZO) has been widely used in thin‐film transistors (TFTs), its application as an active switching medium in resistive random‐access memory (RRAM) has remained relatively less explored, particularly for vertical RRAM (VRRAM) architectures. Here, we report an ALD‐IGZO VRRAM platform in which the IGZO switching layer is conformally formed along vertical sidewalls, providing excellent step coverage and atomic‐level compositional controllability for uniform and reliable switching. This platform realizes a compact dual‐mode device, where the volatile switching mode before forming serves as the reservoir layer and the nonvolatile switching mode after forming functions as the readout layer. Leveraging these coexisting dynamics, we implement a fully hardware‐based reservoir computing (RC) system with a wide architecture by operating multiple sub‐reservoirs in parallel under distinct volatility conditions, achieving enhanced nonlinear mapping and feature separation. The proposed 2F ALD‐IGZO VRRAM wide RC achieves 91.3% accuracy on the MNIST dataset with an estimated energy consumption of 3.70 nJ per classification, offering a scalable route toward learning‐capable in‐memory neuromorphic computing.

## Graphical abstract

A conformal ALD‐IGZO vertical RRAM integrates volatile and nonvolatile switching within a compact 2F architecture. Before forming, tunable volatile dynamics provide fading‐memory reservoir states, while after forming, stable multilevel conductance modulation enables hardware readout. This dual‐mode platform realizes fully hardware‐based wide reservoir computing with parallel sub‐reservoirs, achieving efficient MNIST recognition and scalable in‐memory neuromorphic computing.

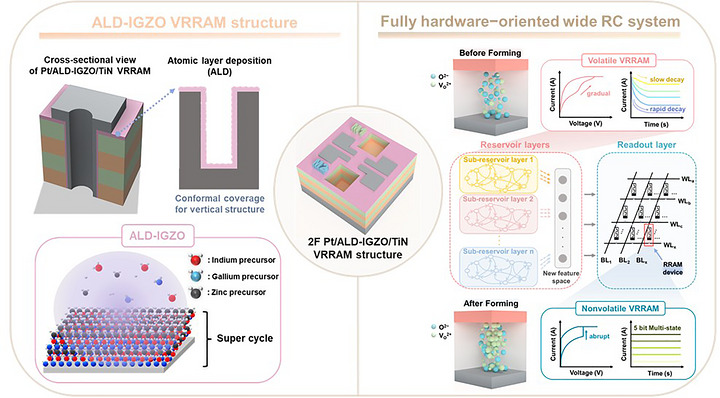

## 1 Introduction

**FIGURE 1:**
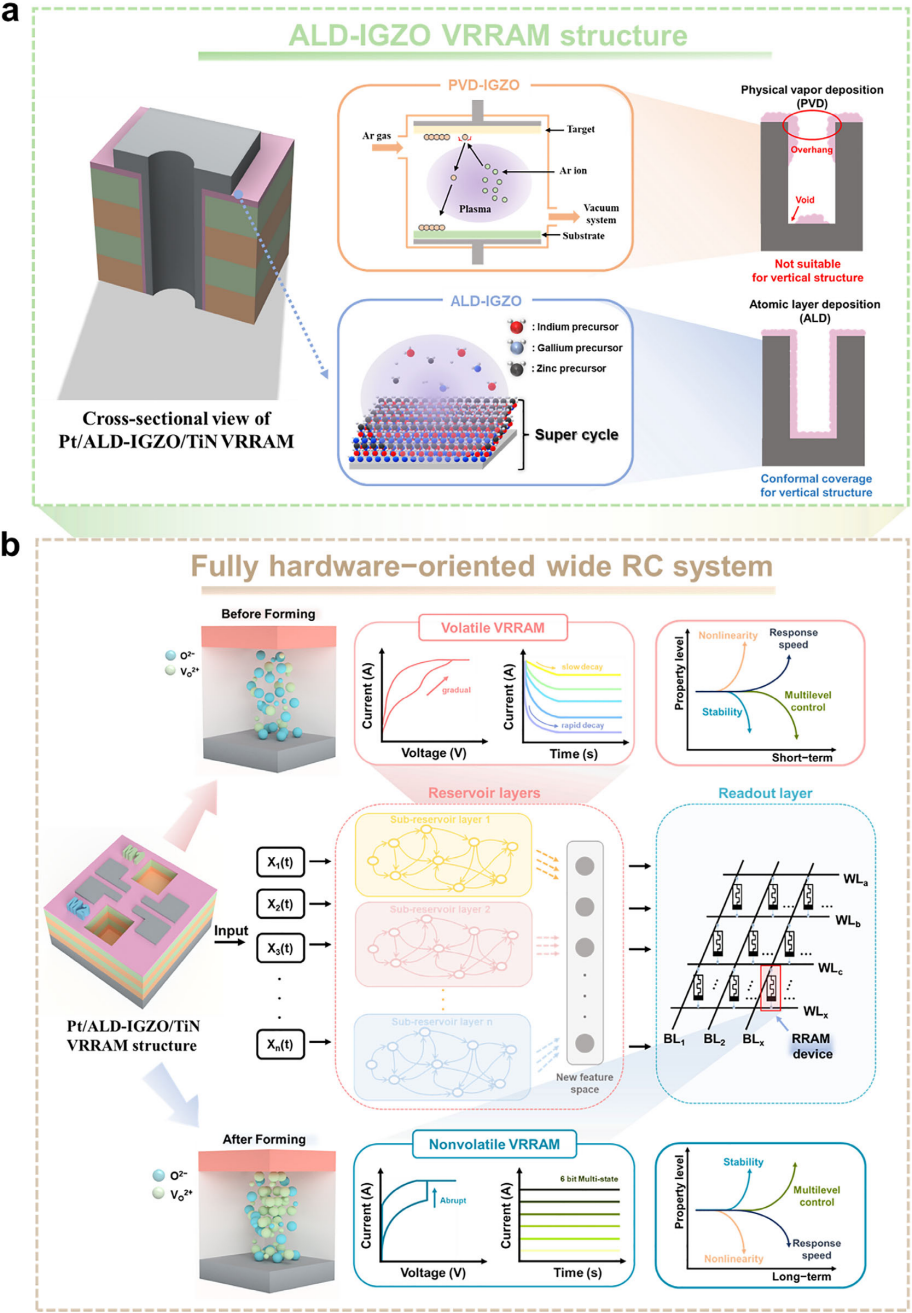
a) Comparison of PVD‐ and ALD‐IGZO films showing the superior conformality of ALD for vertical integration. b) Concept of the fully hardware‐oriented wide RC system using a 2F ALD‐IGZO VRRAM, where volatile operation before forming and nonvolatile operation after forming act as the reservoir and readout layers.

Artificial intelligence (AI) and neuromorphic computing have driven remarkable advances in data‐intensive technologies such as pattern recognition, signal processing, and autonomous systems [1, 2, 3]. However, conventional computing architectures based on the von Neumann model suffer from severe bottlenecks between the memory and the processing units, leading to high power consumption and limited scalability during large‐scale data processing [4, 5]. These limitations have accelerated the exploration of memory‐centric computing paradigms, where computation and storage are co‐located to achieve parallel and energy‐efficient information processing [6, 7, 8, 9]. Among various approaches, reservoir computing (RC) has emerged as an efficient neuromorphic framework for dynamic information processing [10, 11, 12]. An RC system typically consists of three components: an input layer, a nonlinear and dynamic reservoir layer, and a readout layer. The reservoir layer transforms temporal inputs into high‐dimensional states by leveraging intrinsic device dynamics, while only the readout layer requires simple linear regression training [13, 14, 15]. Owing to its simplified learning mechanism and strong adaptability, RC has demonstrated excellent performance in time‐series tasks such as pattern recognition, speech analysis, and chaotic signal prediction [16, 17]. Recently, hardware‐based RC systems have garnered increasing attention because of their superior energy efficiency and integration potential compared with software‐based implementations [18, 19, 20]. In such systems, volatile devices are used for short‐term dynamics in the reservoir layer, and nonvolatile devices store long‐term weights in the readout layer. However, most reported hardware RCs have relied on independent devices for each layer or hybrid configurations combining hardware reservoirs with software‐based readouts, resulting in increased fabrication complexity, power consumption, and limited scalability [21, 22, 23]. Realizing a fully hardware‐based RC system that embodies both short‐term and long‐term memory characteristics within a single platform remains a key challenge [24]. In parallel, enhancing the computational diversity of reservoirs has become an essential direction for improving RC performance. Conventional single‐reservoir structures with uniform device dynamics limit the system's nonlinear mapping capability. To overcome this, the concept of wide RC, where multiple sub‐reservoirs operate in parallel under distinct volatility conditions, has been proposed [25, 26]. Wide RC architectures enable simultaneous multi‐feature extraction and richer temporal representations, thereby improving classification accuracy and generalization. However, most reported wide RC implementations are confined to planar geometries or require additional control gates and external stimuli, increasing system complexity and power consumption. Although indium‐gallium‐zinc oxide (IGZO) has been widely adopted in thin‐film transistor (TFT) technologies because of its high mobility and uniformity, its application in resistive random‐access memory (RRAM) remains relatively unexplored. The majority of IGZO research has focused on planar oxide semiconductors for display technologies, while only a limited number of studies have investigated IGZO as an active switching layer in RRAM devices. This gap becomes even more pronounced in 3D vertical RRAM (VRRAM) architectures, where conformal deposition and compositional control play critical roles in determining device performance and reliability. In particular, realizing uniform and defect‐engineered IGZO films along vertical sidewalls is essential yet challenging, and previous works relying on sputtered IGZO have often suffered from poor step coverage and inconsistent switching characteristics. Therefore, establishing a reliable IGZO‐based VRRAM platform requires not only structural innovation but also process‐level control to stabilize the switching behavior. Developing a 3D vertically integrated wide RC system with diverse and tunable dynamics is therefore a critical step toward scalable and efficient neuromorphic computing. To realize such a vertically integrated neuromorphic framework, it is essential to secure both material‐level conformality and device‐level functional diversity. Figure 1a compares the conformal characteristics of IGZO films deposited by physical vapor deposition (PVD) and atomic layer deposition (ALD) within a VRRAM structure, highlighting the superiority of ALD in achieving uniform sidewall coverage suitable for 3D stacking [27, 28, 29, 30]. Unlike conventional PVD techniques widely employed in industry, ALD utilizes a layer‐by‐layer growth mechanism, enabling precise compositional control and achieving highly uniform thin films. In this experiment, plasma‐enhanced atomic layer deposition (PEALD) was employed among various ALD techniques [31]. Unlike thermal ALD, PEALD utilizes plasma‐activated chemical reactions, enabling low‐temperature deposition. These characteristics are particularly advantageous for vertically stacked VRRAM structures, where minimizing thermal damage during film deposition is essential. Building on this structural advantage, Figure 1b illustrates the concept of the proposed fully hardware‐oriented wide RC system based on a two‐layer (2F) ALD‐IGZO VRRAM, in which the volatile behavior before forming and the nonvolatile switching after forming are integrated to emulate short‐term and long‐term memory functions within a single compact device. In this study, we present a fully hardware‐oriented wide RC system based on ALD‐IGZO VRRAM [32, 33, 34]. The 2F vertically stacked VRRAM structure is designed to utilize the device's volatile behavior before forming as the reservoir layer and its nonvolatile switching after forming as the readout layer, thereby integrating short‐term and long‐term memory functionalities within a single device. The device before forming exhibits fading‐memory dynamics originating from charge trapping and detrapping, enabling effective temporal information processing, whereas after forming, it achieves trainable long‐term weight modulation through the incremental step pulse with verification algorithm (ISPVA), which is suitable for implementing learning functions in the readout network [35, 36]. Furthermore, the proposed system exhibits key synaptic behaviors such as excitatory postsynaptic current (EPSC) and paired‐pulse facilitation (PPF), confirming its capability to emulate both short‐ and long‐term plasticity in hardware [37, 38, 39]. By employing the volatile and nonvolatile VRRAM layers as the reservoir and readout components, respectively, the system performs fully hardware‐oriented pattern‐recognition tasks on the MNIST and Fashion‐MNIST datasets, achieving high classification accuracy with low energy consumption. This study provides a scalable and energy‐efficient platform for next‐generation neuromorphic computing, demonstrating the feasibility of integrating multiple dynamic functionalities within a single vertically stacked device.

## 2 Results and Discussion

### 2.1 ALD‐IGZO Integration in 2F VRRAM: Composition Control and Structural Analysis

**FIGURE 2:**
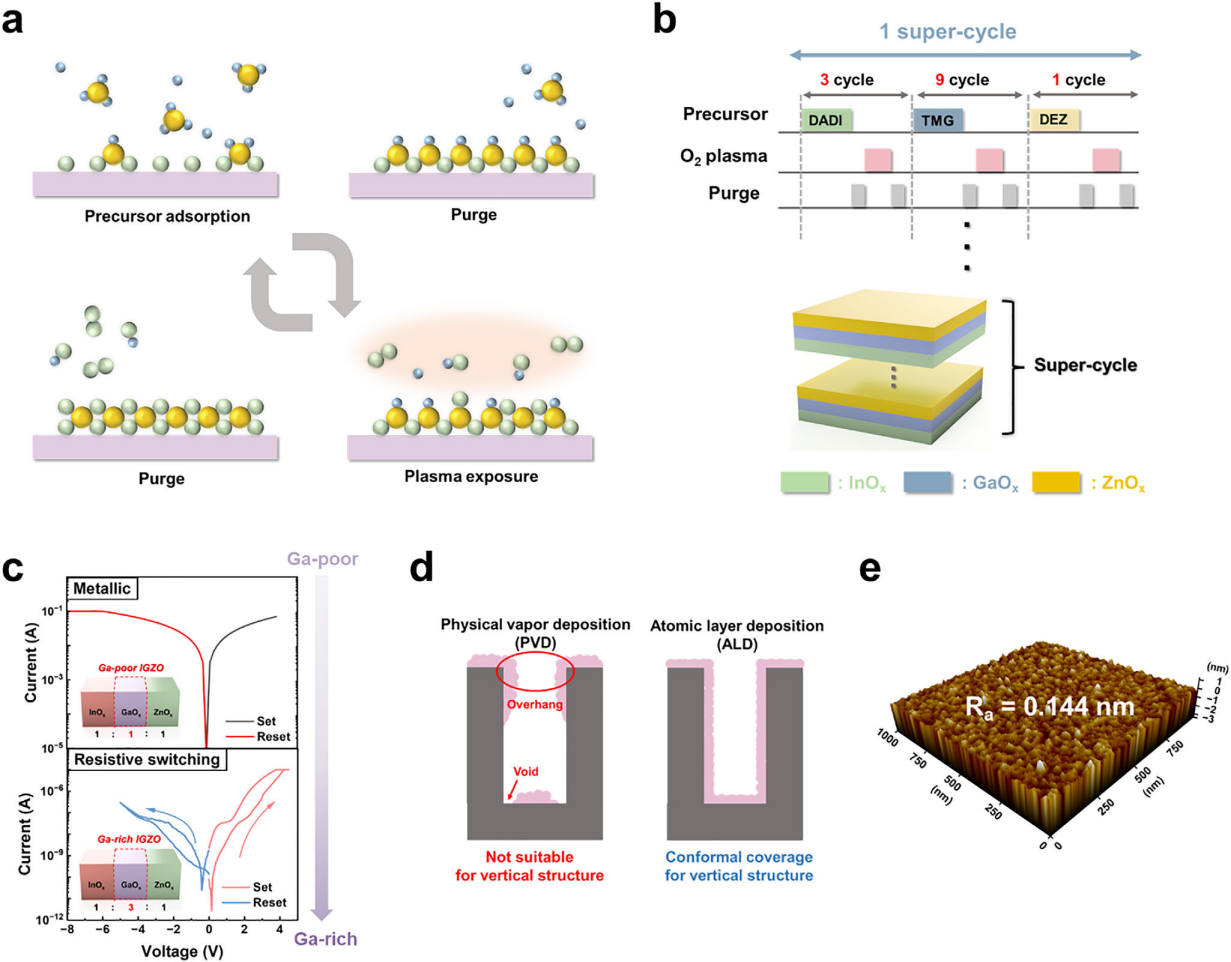
a) Simplified schematic of a typical PEALD process mechanism. b) Schematic illustration of ALD‐IGZO deposited with supercycle. c) Metallic‐to‐resistive switching transition enabled by Ga‐rich IGZO (1:3:1) compared to Ga‐poor IGZO (1:1:1). d) Comparison of step coverage between ALD and PVD in a vertical structure. e) AFM measurement of IGZO by ALD process.

**FIGURE 3:**
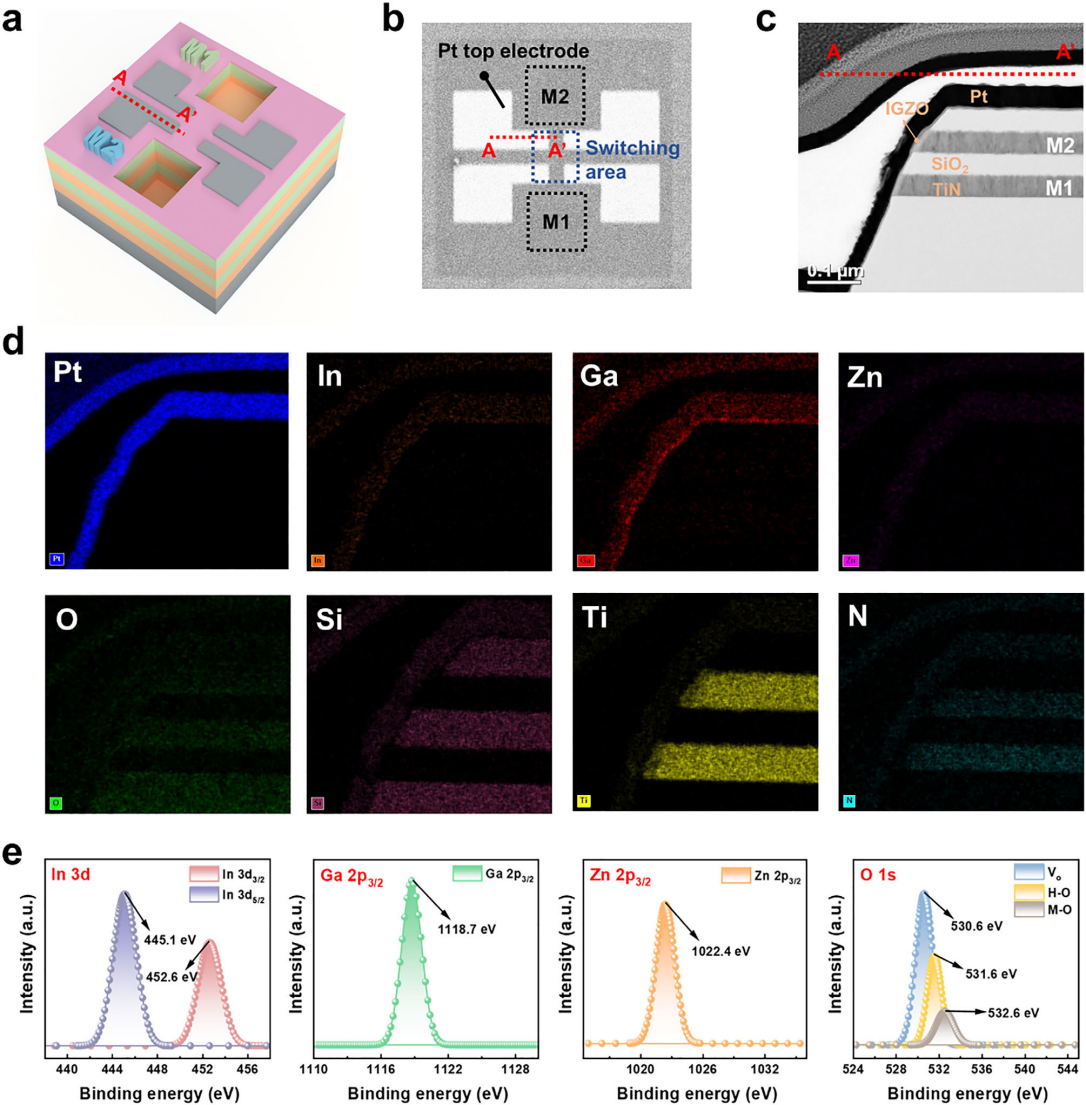
a) 3D schematic diagram illustrating the overall structure of the device. b) Top‐view image obtained using SEM. c) Cross‐sectional TEM image of the device. d) EDS elemental mapping images of Pt, In, Ga, Zn, O, Si, Ti, and N. e) The XPS analysis of the ALD‐IGZO switching layer.

Figure 2 summarizes the ALD‐based IGZO deposition process, the compositional optimization strategy, and the advantages of ALD for vertical VRRAM integration. Figure 2a illustrates a simplified PEALD cycle, consisting of precursor adsorption, a first purge, plasma exposure, and a second purge step, which are sequentially repeated to form each metal‐oxide sub‐layer [40]. Figure 2b shows the super‐cycle architecture used for IGZO deposition, consisting of InO_x_, GaO_x_, and ZnO_x_ sub‐cycles. Based on the measured growth‐per‐cycle (GPC) values for each oxide (0.08 nm/cycle for InO_x_, 0.10 nm/cycle for GaO_x_, and 0.25 nm/cycle for ZnO_x_), the sub‐cycle ratio was tuned to In:Ga:Zn = 3:9:1 to achieve the target IGZO composition of 1:3:1. The PEALD process was performed at 150°C and 10 mTorr using (3‐(dimethylamino) propyl) dimethylindium (DADI), trimethylgallium (TMG), and diethylzinc (DEZ) as the respective metal precursors [29, 41]. Prior to fabricating VRRAM devices, a systematic single‐cell test was conducted using IGZO films with varying thicknesses (3–7 nm) and In:Ga:Zn compositions, as summarized in Figure S2a. Figure 2c presents the evolution of the VRRAM switching characteristics as a function of Ga content in the IGZO composition. Devices employing IGZO compositions other than In:Ga:Zn = 1:3:1 (devices 1–10) exhibit metallic‐like I‐V characteristics without resistive switching, as shown in Figure S2b. This metallic conduction originates from the excessive formation of intrinsic oxygen vacancies (V_O_) in Ga‐poor IGZO, arising from relatively weak metal‐oxygen bonding, which results in a high free‐electron concentration and suppresses the formation of a stable off state. In contrast, Ga‐rich IGZO devices with an In:Ga:Zn ratio of 1:3:1 (devices 11 and 12) show a clear transition to stable resistive switching, as shown in Figure S2c. The increased Ga incorporation strengthens the metal‐oxygen bonding network, thereby suppressing the spontaneous generation of V_O_ and reducing the background carrier density. This stabilization of the insulating initial state enables field‐driven modulation of V_O_ under electrical bias, resulting in well‐defined set and reset processes and a pronounced ON/OFF switching behavior suitable for VRRAM operation. Based on these results, the 5 nm‐thick Ga‐rich IGZO device 11 was selected as the switching layer for subsequent VRRAM fabrication. Figure 2d compares the conformality of IGZO films deposited by PVD and ALD in a vertical structure. While PVD exhibits limited sidewall coverage, ALD provides uniform deposition along the vertical surfaces, which is essential for stacked VRRAM architectures. Figure 2e shows the AFM analysis of the ALD‐grown IGZO thin film, exhibiting a highly smooth morphology with a roughness average (R_a_) of approximately 0.144 nm, demonstrating the excellent thin‐film uniformity achieved by ALD. Figure 3a presents a 3D schematic diagram illustrating the overall structure of the Pt/ALD‐IGZO/TiN VRRAM device. Figure 3b shows a top‐view scanning electron microscopy (SEM) image of the fabricated device, revealing the circular contact‐hole pattern with a diameter of approximately 10 µm. The contact holes are filled with Pt pillar electrodes that act as the top electrodes of the VRRAM cell. The switching region, highlighted by the blue dashed lines, is located along the sidewall of the etched hole, oriented vertically with respect to the substrate. The TiN layers (1F and 2F) are separated by SiO_2_ insulating layers, functioning as the bottom electrodes within the stacked configuration. To further analyze the structural geometry of the holes, Figure S3a shows a cross‐sectional SEM image confirming the hole diameter, while Figure S3b presents the etched sidewall slope. In addition, Figure S3c provides a cross‐sectional schematic summarizing both the hole diameter and sidewall angle. These results indicate that the etching process produced a hole structure with a smooth and uniform sidewall profile, suitable for conformal film growth by ALD. Figure 3c displays a cross‐sectional transmission electron microscopy (TEM) image of the device along the A–A′ direction, clearly showing that the TiN planar electrodes are separated by the SiO_2_ interlayer dielectric. In addition, the 5 nm‐thick ALD‐IGZO film conformally coats the sidewall of the contact hole, demonstrating the excellent step coverage achieved through the ALD process. Figure 3d shows the energy‐dispersive spectroscopy (EDS) elemental mapping images obtained from the cross‐section of the Pt/ALD‐IGZO/TiN VRRAM device, confirming the elemental distribution of Pt, In, Ga, Zn, O, Si, Ti, and N within the stacked structure. Figure 3e shows the X‐ray photoelectron spectroscopy (XPS) of the IGZO thin film. The In 3d core‐level spectrum exhibits two distinct peaks at 445.1 eV (In 3d_5/2_) and 452.6 eV (In 3d_3/2_), verifying the fully oxidized indium states within the amorphous IGZO matrix. The Ga 2p_3/2_ and Zn 2p_3/2_ peaks appear at 1118.7 and 1022.4 eV, respectively, consistent with Ga^3+^‐O and Zn^2+^‐O bonding environments typically observed in oxide semiconductors. The quantified cation composition of the ALD‐IGZO film was 4 at.% In, 13 at.% Ga, and 3 at.% Zn, confirming that the intended In:Ga:Zn stoichiometric ratio of 1:3:1 was successfully achieved in the deposited film. The O 1s spectrum was deconvoluted into three components centered at 530.6, 531.6, and 532.6 eV, corresponding to non‐lattice oxygen associated with V_O_, hydroxyl‐related defect states (H‐O), and metal‐oxygen lattice bonding (M‐O), respectively. Notably, the V_O_ component near 531 eV dominated the O 1s spectrum, indicating the presence of a considerable concentration of oxygen‐vacancy states within the IGZO thin film. These features collectively verify the chemically stable amorphous structure of the ALD‐IGZO and highlight the crucial role of oxygen‐related defects in governing the electrical behavior of the device.

### 2.2 Volatile Switching and Short‐Term Dynamics for Reservoir Operation

**FIGURE 4:**
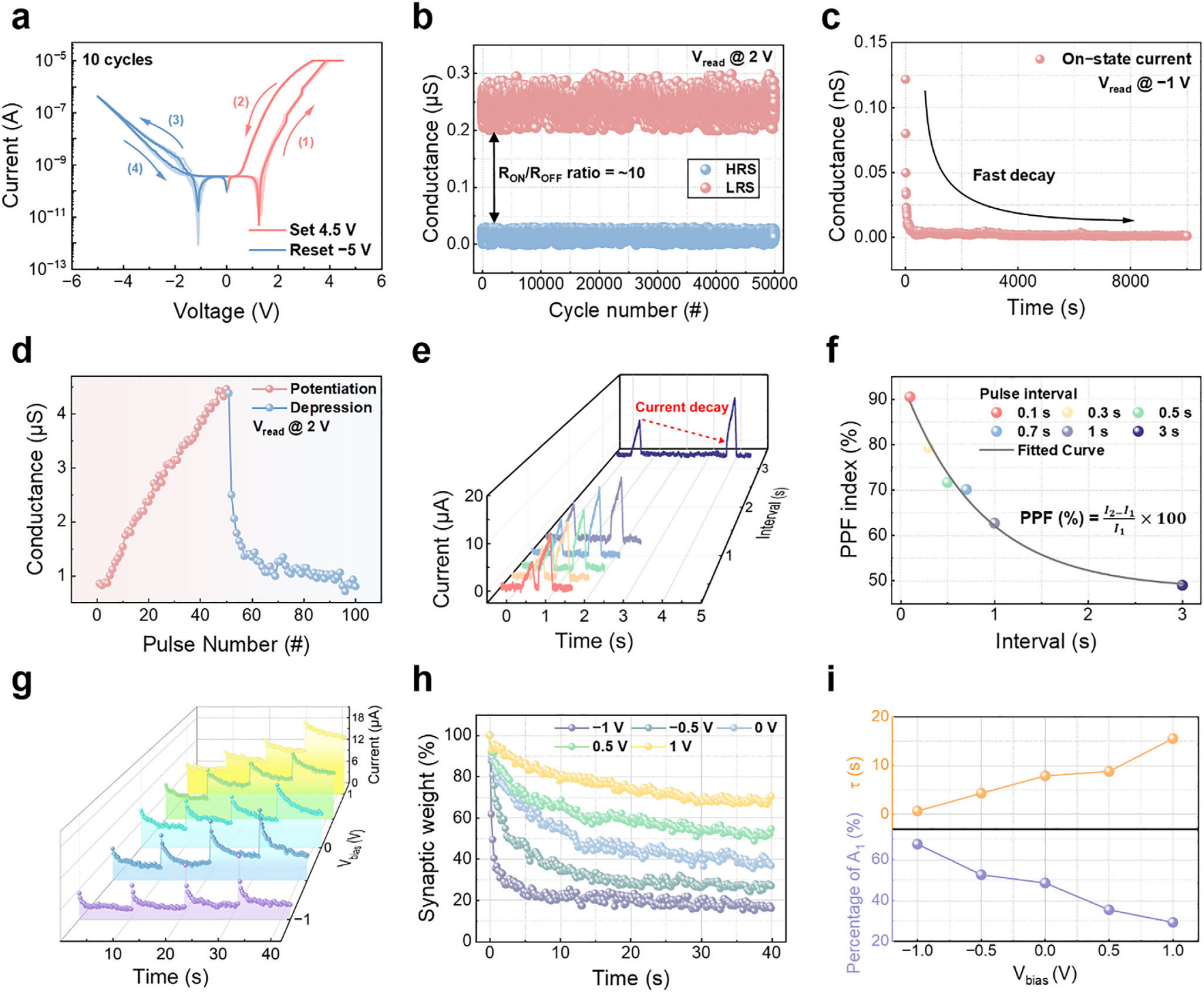
Electrical characteristics of the 1F Pt/ALD‐IGZO/TiN VRRAM cell exhibiting volatile behavior before the forming process. a) Consecutive *I*–*V* curves measured over 10 sweep cycles. b) Pulse endurance characteristics of the volatile switching behavior measured over 50 000 consecutive cycles at a read voltage of 2 V. c) Decay behavior of the on‐state current monitored at a read voltage of −1 V for 10 000 s. d) P&D characteristics under consecutive pulse stimulation. e) PPF behavior measured under different pulse interval times. f) PPF index as a function of interval time ranging from 0.1 to 3 s. g) Memory decay behavior of the device under different V_bias_ conditions. h) Synaptic weight decay behavior of the device measured under various bias voltages (−1 to 1 V) over 40 s. i) Extracted decay time constants (τ_1_) and percentage of A_1_ as a function of applied V_bias._

This conformal ALD‐IGZO film along the vertical sidewall enables uniform relaxation before forming, which is essential for reservoir behavior. Therefore, we first characterize the volatile response before forming, as presented in Figure 4 [42, 43]. To analyze the electrical characteristics of the 1F Pt/ALD‐IGZO/TiN VRRAM cell, a voltage bias was applied to the top Pt electrode while grounding the bottom TiN electrode. Figure 4a shows the consecutive *I*–*V* characteristics of the 1F VRRAM cell measured over 10 sweep cycles before the forming process. When a positive bias of 4.5 V was applied, the current increased during the dual voltage sweep and reached a compliance current (CC) of 10 µA, as indicated in process (1) of Figure 4a. This compliance limit prevented permanent breakdown and induced a transition to a higher current state, as shown in process (2), corresponding to the resistive switching behavior from a high‐resistance state (HRS) to a low‐resistance state (LRS). Conversely, when a negative bias of −5 V was applied, the current decreased during the sweep, as seen in processes (3) and (4), indicating a reset process that switched the device back from the LRS to the HRS. A similar gradual current change was also observed in the 2F VRRAM cell, as presented in Figure S5a, confirming consistent switching characteristics across both layers. To further investigate the electrical characteristics across different layers in the vertically stacked VRRAM structure, additional cell‐to‐cell measurements were performed on ten devices for both the 1F and 2F layers before the forming process, as shown in Figure S6. The cell‐to‐cell HRS and LRS resistance distributions are statistically summarized in the box plots shown in Figure S7a, while the cumulative probability distributions are presented in Figure S7b. Although differences in the absolute resistance levels were observed between the 1F and 2F layers, both layers exhibited clearly distinguishable HRS and LRS states with comparable volatile switching characteristics and low device‐to‐device variation. Nevertheless, the overall volatile switching behavior remained consistent throughout the vertically stacked structure. The observed resistance shift is likely associated with the tapered geometry of the etched vertical hole rather than a change in the switching mechanism. To evaluate the endurance of the volatile resistive switching behavior, extended pulse endurance measurements over 50 000 consecutive operating cycles were performed for both the 1F and 2F VRRAM cells. As shown in Figure 4b, the HRS and LRS of the 1F cell remain clearly distinguishable throughout 50 000 consecutive cycles under repeated 2 V pulse stimulation, while maintaining a R_ON_/R_OFF_ ratio of approximately 10 without noticeable degradation. Magnified views of the initial (cycles 1–100), intermediate (cycles 24 951–25 050), and final (cycles 49 901–50 000) cycling regions are presented in Figure S4a. Furthermore, the conductance modulation window (ΔG) was evaluated as a function of cycle number, as shown in Figure S4b. Each data point represents the mean ΔG calculated over 100 consecutive cycles, and no systematic decrease from the overall mean value of 0.222 µS was observed throughout the endurance measurement. These results demonstrate the excellent endurance and reproducibility of the volatile switching characteristics under repeated operation, which are essential for reliable reservoir computing applications. The 2F cell also maintained stable and clearly distinguishable conductance states over 50 000 consecutive cycles, confirming reliable volatile switching behavior in the vertically stacked structure, as shown in Figure S5b. Figure 4c presents the decay of the on‐state current monitored at a read voltage of −1 V for 10 000 s [44, 45]. The rapid decay in current reflects the volatile nature of the 1F cell, suggesting that the Pt/ALD‐IGZO/TiN VRRAM can operate as a short‐term memory (STM) element. Comparable current levels and short‐term behaviors were also observed in the 2F cell, as shown in Figure S5c. Such dynamic resistive switching and fading‐memory behavior are essential features for the reservoir layer in hardware‐based RC systems. In particular, the volatile response enables nonlinear temporal information encoding and multiple relaxation time constants, allowing the device to emulate physical reservoir nodes that process input signals across different timescales. The vertical stacking further provides structural scalability for constructing hierarchical multi‐reservoir layers, which can enhance feature diversity and computing capacity in wide RC architectures [46]. To verify the synaptic reliability of the 1F Pt/ALD‐IGZO/TiN VRRAM cell, potentiation and depression (P&D) characteristics were evaluated using consecutive electrical pulse stimulations. The pulse conditions used for the measurements are presented in Figure S8a, where the set, reset, and read pulses were applied at 3 V for 0.05 s, −2 V for 0.05 s, and 2 V for 0.1 s, respectively. As illustrated in Figure 4d, the 1F cell exhibited a gradual increase in conductance during the potentiation, followed by a progressive decrease during the depression, demonstrating smooth and controllable analog synaptic weight modulation. This conductance evolution remained stable and continuous over 50 consecutive pulse stimulations, confirming reliable analog switching behavior. Importantly, the 2F Pt/ALD‐IGZO/TiN VRRAM cell exhibited comparable P&D characteristics under identical pulse conditions, as shown in Figure S5d, confirming consistent synaptic behavior across the vertically stacked layers. To further assess the reproducibility of the synaptic operation, 10 consecutive P&D cycles were conducted, as depicted in Figure S8b. Although slight variations in conductance values were observed between cycles, the overall modulation trend remained consistent, indicating that a 1F VRRAM cell can stably reproduce analog synaptic behavior suitable for neuromorphic applications [47, 48]. In addition, the tunability of the synaptic weight modulation was examined under various pulse conditions, as shown in Figure S9. Figure S9a presents the pulse scheme used for P&D measurements under different pulse numbers. Figure S9b shows the corresponding conductance modulation behavior, where increasing the number of applied set/reset pulses expanded the conductance window, indicating that repeated stimulations promote gradual potentiation. Figure S9c illustrates the pulse scheme employed for P&D under varying pulse widths. Figure S9d displays the resulting conductance modulation, demonstrating that longer pulse widths induce greater conductance increases during potentiation. These results confirm that the input energy per pulse strongly influences synaptic weight updates and collectively demonstrate that the Pt/ALD‐IGZO/TiN VRRAM cell enables flexible and controllable analog weight modulation through pulse condition adjustments, ensuring stable operation for reliable synaptic functionality. To investigate the short‐term plasticity (STP) behavior, PPF measurements were conducted by applying two consecutive pulses at different interval times (Δt = 0.1, 0.3, 0.5, 0.7, 1, and 3 s) [49, 50]. PPF, a representative form of short‐term synaptic plasticity observed in biological synapses, describes the phenomenon in which the response to the second pulse becomes stronger as the interval between two pulses decreases. Figure 4e shows the current responses measured at six different intervals, where the post‐pulse current (I_2_) consistently exceeds the pre‐pulse current (I_1_), indicating a clear facilitation effect of the volatile memristor. The PPF index was calculated using the following equation:

(1) $$PPFindex=\frac{I_{2}-I_{1}}{I_{1}}\times 100\left(\%\right)$$

where I_1_ and I_2_ represent the average current values of the pre‐ and post‐pulses, respectively. As shown in Figure 4f, the PPF index decreases exponentially with increasing interval time, indicating that the influence of the pre‐pulse gradually weakens as the interval becomes longer. This behavior closely mimics the STM dynamics of biological synapses, where memory retention naturally decays over time [51, 52, 53]. As illustrated in Figure 4g, the bias‐dependent decay behavior clearly demonstrates the tunable volatility of the 1F Pt/ALD‐IGZO/TiN VRRAM. When the applied bias increased from −1 to 1 V, the current decay rate became slower, indicating that the relaxation time (τ) increases with the electric field strength. At −1 V, the current rapidly decayed to its steady‐state level, exhibiting strong volatility, whereas at 1 V, the current maintained a more stable state throughout the 40 s measurement period. To further visualize the temporal fading process, Figure 4h presents the normalized current evolution, representing the transient synaptic weight under various bias conditions, derived from the decay curves in Figure 4g. As the bias voltage increased, the normalized current decayed more slowly, highlighting the bias‐dependent suppression of volatility. This normalized representation allows a clear comparison of fading behaviors, emphasizing that stronger biases enhance stability by extending the relaxation time. The relaxation dynamics were quantitatively analyzed using a single‐exponential decay model expressed as:

(2) $$I\left(t\right)=A_{1}\exp\left(-\frac{t}{\tau }\right)+I_{0}$$

where I(t) is the transient current at time t, A_1_ represents the amplitude of the decay component, I_0_ is the steady‐state current, and τ denotes the decay time constant. The extracted parameters are summarized in Figure 4i, and detailed decay curves for each bias condition are provided in Figure S10a–e. As the bias increased from −1 to +1 V, τ increased from 0.66 to 15.5 s, indicating slower carrier relaxation and enhanced temporal retention under stronger electric fields. In contrast, A_1_ decreased from 67.6% to 29.2%, reflecting reduced current variation and more stable conductive paths. These results confirm that higher bias conditions extend the relaxation process and suppress volatility. This provides precise electrical control of STM dynamics for RC applications. To further evaluate the cycle‐to‐cycle uniformity of the volatile characteristics, repeated decay measurements were performed over 50 consecutive cycles at a read bias of −1 V, and the results are summarized in Figure S11. As shown in Figure S11a, the decay responses exhibit highly reproducible relaxation behavior with minimal cycle‐to‐cycle variation. The extracted decay time constant (τ) remained stable across repeated measurements, yielding an average value of 0.604 s with a coefficient of variation (CV) of 7.93%, as presented in Figure S11b. In addition, the transient response amplitude (ΔI = I_peak_ − I_baseline_) showed a small variation with a CV of 5.23% over 50 cycles, as shown in Figure S11c. These results demonstrate the excellent reproducibility of the volatile dynamics, confirming that the device can generate stable reservoir states for reliable temporal information processing.

**FIGURE 5:**
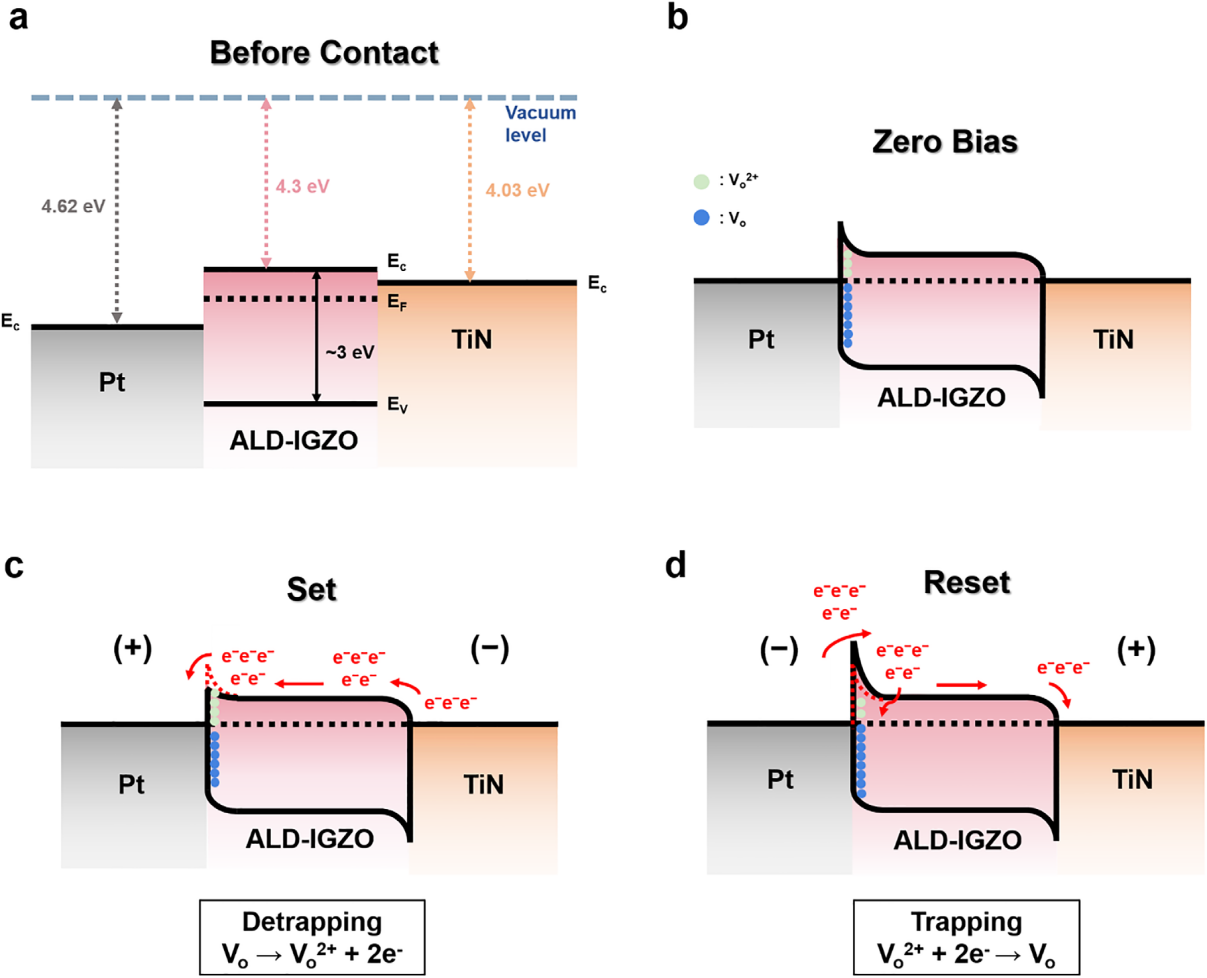
a) Flat band diagram of the Pt/ALD‐IGZO/TiN VRRAM device. b) Energy band diagram under zero bias. c) Energy band diagram under positive bias during the set process. d) Energy band diagram under negative bias during the reset process.

Ultraviolet photoelectron spectroscopy (UPS) measurements were performed on Pt, TiN, Pt/IGZO, and TiN/IGZO reference samples fabricated under the same deposition conditions as the devices to experimentally determine the electrode work functions, as shown in Figure S12 [54]. The measured work functions were 4.62 and 4.03 eV for Pt and TiN, respectively, while Pt/IGZO and TiN/IGZO exhibited nearly identical values of 4.64 and 4.04 eV, indicating that the IGZO layer does not significantly alter the electrode work functions. The UPS‐derived work function of the deposited Pt film (∼4.6 eV) is lower than the typical range of 5.3–5.7 eV reported for atomically clean Pt surfaces [55, 56]. This deviation is likely associated with the process‐dependent surface condition of the deposited Pt film, including surface adsorbates or residual contamination, surface roughness, crystallographic texture, and uncertainty in determining the secondary‐electron cutoff. In particular, carbon adsorption on Pt has been reported to reduce its work function [57]. Accordingly, the measured value should be regarded as the effective surface work function of the deposited Pt film under the present fabrication and UPS measurement conditions, rather than as the intrinsic work function of an atomically clean Pt single crystal. Importantly, the measured Pt work function remains 0.59 eV higher than that of TiN, preserving the relative electrode asymmetry used to construct the energy band diagram. Based on these experimentally determined work functions together with the electron affinity (∼4.3 eV) and bandgap (∼3 eV) of IGZO, the energy band diagram shown in Figure 5a was constructed. As shown in Figure 5a, the Schottky barrier at the Pt/ALD‐IGZO interface is higher than that at the ALD‐IGZO/TiN interface, resulting in distinct conduction mechanisms depending on the polarity of the applied bias. As illustrated in Figure 5b, at equilibrium, the Fermi level (E_F_) lies closer to the conduction band near the TiN bottom electrode, leading to a relatively lower barrier at the ALD‐IGZO/TiN interface while maintaining a higher barrier at the Pt/ALD‐IGZO interface. V_O_ are distributed throughout the ALD‐IGZO layer, and a portion of them are ionized (V_O_^2+^), forming donor‐like energy states. This configuration corresponds to the stage before forming, where volatile switching arises due to transient electron trapping and detrapping, resulting in temporary current retention followed by gradual decay. Under a positive bias applied to the Pt electrode, as illustrated in Figure 5c, the effective Schottky barrier at the Pt/ALD‐IGZO interface decreases, facilitating electron injection from TiN. The strong electric field drives oxygen ions (O^2^^−^) toward the bottom electrode, while some V_O_ are ionized (V_O_ → V_O_^2+^ + 2e^−^), increasing donor states and electron density in the ALD‐IGZO layer. Consequently, the current rises through tunneling and thermionic emission, switching the device from HRS to LRS. This behavior indicates a barrier‐controlled set mechanism rather than filament formation. As shown in Figure 5d, applying a negative bias to the Pt electrode reverses the electric field direction, causing O^2^^−^ to drift toward the Pt/ALD‐IGZO interface. The previously ionized V_O_^2+^ recombine with electrons to form neutral V_O_, thereby restoring the interfacial Schottky barrier height and suppressing electron transport. As a result, the device returns from LRS to HRS. Ultimately, the reversible ionization and neutralization of oxygen vacancies (V_O_ ⇌ V_O_^2+^ + 2e^−^) periodically modulate the Schottky barrier height, giving rise to gradual volatile switching behavior [58]. To further verify the proposed Schottky‐barrier‐modulation mechanism, temperature‐dependent electrical measurements were performed from 25 to 125°C, as shown in Figure S13a [59, 60, 61, 62]. The current level increased with increasing temperature, indicating thermally activated carrier transport. According to the thermionic emission model, the current transport through the metal/semiconductor interface can be described as:

(3) $$I=AA^{*}T^{2}\exp\left(-\frac{q\emptyset_{B,eff}}{k_{B}T}\right)$$

where *A* is the device area, A* is the Richardson constant, *q* is the elementary charge, *k_B_* is the Boltzmann constant, *T* is the absolute temperature, and ∅_*B*, eff_ is the effective Schottky barrier height. Rearranging the equation yields

(4) $$\ln\left(\frac{I}{T^{2}}\right)=\;\ln\left(AA^{*}\right)-\frac{q\emptyset_{B,eff}}{k_{B}T}\frac{1}{T}$$

Therefore, the effective Schottky barrier height can be extracted from the slope of the Arrhenius plot of $\phi_{B,eff}=\;-\frac{k_{B}}{q}\times slope$ versus 1/*T* according to

(5) $$\emptyset_{B,eff}=-\frac{k_{B}}{q}\times slope$$

As shown in Figure S13b, the Arrhenius plots exhibit good linearity for both the HRS and LRS states measured at the same read voltage of 2 V. The extracted effective Schottky barrier heights were 0.29 and 0.158 eV for the HRS and LRS, respectively. The significantly reduced barrier height in the LRS indicates enhanced electron injection through thermionic emission, which is consistent with the proposed barrier‐lowering process during the SET operation. These results experimentally support the volatile switching mechanism governed by reversible oxygen‐vacancy ionization and Schottky barrier modulation at the Pt/ALD‐IGZO interface. This dynamic barrier modulation underlies the STM characteristics of the device. Furthermore, the experimentally extracted effective Schottky barrier heights (0.29 eV for HRS and 0.158 eV for LRS) confirm that the volatile switching behavior originates from Schottky‐barrier modulation and thermionic‐emission transport rather than filament formation.

### 2.3 Nonvolatile Switching and Synaptic Weight Modulation for Readout Layer

**FIGURE 6:**
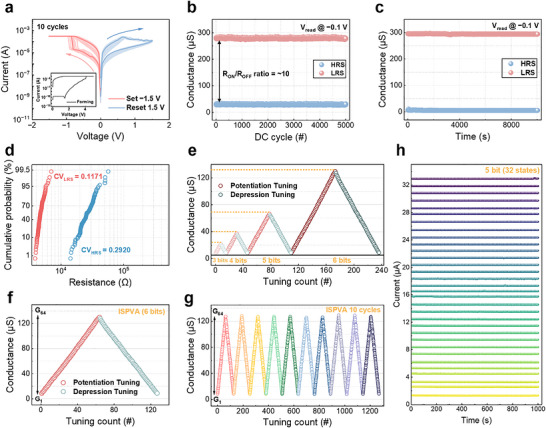
Electrical characteristics of the 1F Pt/ALD‐IGZO/TiN VRRAM cell exhibiting nonvolatile behavior after the forming process. a) Consecutive *I*–*V* curves measured over 10 sweep cycles including the forming process. b) Nonvolatile switching endurance characteristics measured over 5000 consecutive cycles at a read voltage of −0.1 V. c) Retention characteristics obtained at a read voltage of −0.1 V for 10 000 s. d) CV values of the HRS and LRS obtained from consistent DC switching cycles. e) 3, 4, 5, and 6‐bit P&D curves measured using ISPVA. f) 6‐bit conductance modulation characteristics using ISPVA. g) Reproducibility of 6‐bit ISPVA operation over 10 consecutive cycles. h) Retention characteristics of 5‐bit current states measured for 1000 s at −0.1 V.

We next trigger forming to switch from relaxation‐dominated volatile behavior to filamentary nonvolatile switching suitable for the readout layer. Unlike the volatile switching behavior observed before forming, the 1F Pt/ALD‐IGZO/TiN VRRAM cell exhibited stable nonvolatile resistive switching after forming. Figure 6a shows the *I*–*V* characteristics of the 1F VRRAM cell, with the inset displaying the forming *I*–*V* curve that exhibits an abrupt current increase at approximately 5 V. A voltage bias was applied to the top Pt electrode, while the bottom TiN electrode was grounded. The forming process was performed by sweeping the voltage from 0 to 5 V under a CC of 1 mA, during which a sudden current jump indicated the initiation of conductive path formation within the ALD‐IGZO layer [63, 64]. Under this electric field, O^2−^ migrated toward the top electrode, creating V_O_^2+^ near the bottom electrode, which served as seeds for subsequent filament growth, as schematically illustrated in Figure S14a, b, which shows the initial and forming states. After forming, the 1F VRRAM cell exhibited typical bipolar resistive switching characteristics. When a negative bias was applied to the top electrode, the current abruptly increased at −1.5 V with a CC of 0.3 mA, corresponding to the set process, during which conductive filaments were formed through the accumulation of V_O_, transitioning the device from HRS to LRS. Conversely, applying a positive bias of approximately 1.5 V triggered the reset process, where the current decreased sharply as O^2−^ migrated back toward the top electrode and recombined with V_O_^2+^, resulting in partial filament rupture, as shown in Figure S14c, d, which shows the set and reset states. After forming, the switching mechanism of the ALD‐IGZO‐based VRRAM device was dominated by the formation and rupture of conductive filaments driven by O^2−^ migration and V_O_ recombination [65, 66, 67]. Additionally, Figure S15a presents the *I*–*V* characteristics of the 2F cell, which displayed a similar bipolar switching behavior. Although the set window was slightly narrower than that of the 1F cell, the 2F cell maintained uniform and stable nonvolatile switching performance across the vertically stacked structure. To further investigate the electrical characteristics across different layers in the vertically stacked VRRAM structure after the forming process, additional cell‐to‐cell measurements were performed on ten devices for both the 1F and 2F layers, as shown in Figure S16. The cell‐to‐cell HRS and LRS resistance distributions are statistically summarized in the box plots shown in Figure S17a, while the cumulative probability distributions are presented in Figure S17b. Both the 1F and 2F layers exhibited clearly distinguishable HRS and LRS states with comparable nonvolatile switching characteristics and low device‐to‐device variation. Furthermore, the statistical distributions of the two layers showed highly similar resistance levels, indicating excellent layer‐to‐layer uniformity throughout the vertically stacked VRRAM structure. Figure 6b,c show the endurance and retention characteristics of the 1F VRRAM cell. All measurements were conducted at a read voltage of −0.1 V. To further evaluate the reliability of the nonvolatile memory characteristics, the endurance measurement was extended to 5000 consecutive switching cycles. As shown in Figure 6b, the 1F cell maintained clearly distinguishable HRS and LRS states throughout 5000 consecutive switching cycles without significant degradation of the memory window, demonstrating excellent nonvolatile switching endurance. In addition, both resistance states were stably preserved for 10 000 s at room temperature, as shown in Figure 6c, confirming the robust retention characteristics and nonvolatile memory behavior of the device. To further evaluate the thermal stability of the nonvolatile memory characteristics, additional retention measurements were performed at elevated temperatures of 85°C and 125°C, as shown in Figure S18 [68]. Although slight conductance fluctuations were observed as the temperature increased, the HRS and LRS remained clearly distinguishable throughout 10 000 s without significant degradation of the memory window. These results demonstrate that the proposed ALD‐IGZO‐based VRRAM device exhibits excellent high‐temperature retention characteristics and maintains stable nonvolatile memory behavior under elevated‐temperature operating conditions. The 2F cell, as shown in Figure S15b, c, exhibited nearly identical endurance and retention behaviors with comparable conductance levels, demonstrating that the ALD‐IGZO‐based VRRAM maintains robust reliability and data retention even in a vertically stacked configuration. Figure 6d presents the CV values for the HRS and LRS states obtained from consistent DC switching cycles, calculated as 0.2920 and 0.1172, respectively. The relatively low CV values indicate stable resistive switching with well‐defined separation between the two states. Figure S15d also presents the CV results for the 2F cell. Figure 6e displays the P&D curves of the 1F VRRAM cell measured using the ISPVA for 3‐, 4‐, 5‐, and 6‐bit states. Each bit state was adjusted by gradually increasing or decreasing the pulse amplitude in 50 mV steps, with a read voltage of −0.1 V. The ISPVA method produced clearly distinguishable current responses and excellent linearity at each bit level, confirming precise conductance modulation capability. As illustrated in Figure 6f, the 6‐bit P&D characteristics exhibited highly linear conductance modulation behavior, which is advantageous for weight training in RC readout layers. Figure 6g presents 10 consecutive P&D cycles under identical 6‐bit ISPVA conditions, showing minimal variation in current levels and excellent reproducibility [69, 70]. To further evaluate the statistical robustness of the multilevel operation, additional cell‐to‐cell analyses were performed using 10 independently measured VRRAM cells, as shown in Figure S19. Figure S19a presents the 6‐bit potentiation and depression characteristics obtained from 10 independently fabricated VRRAM cells, demonstrating highly reproducible multilevel conductance modulation with negligible cell‐to‐cell variation. Furthermore, the adjacent‐state margin (ΔG/σ_avg_) analysis shown in Figure S19b,c reveal that all conductance states satisfy the ΔG/σ_avg_ > 2 criterion for reliable state separation, confirming the absence of adjacent‐state overlap. The statistical distributions of the conductance states are summarized in Figure S19d,e, from which the average CVs were calculated to be only 1.02% and 1.09% for potentiation and depression, respectively. These results demonstrate that the conductance separation between adjacent states is sufficiently larger than the corresponding cell‐to‐cell variation, thereby ensuring reliable and distinguishable 6‐bit multilevel programmability for hardware synaptic weight storage. Furthermore, Figure 6h demonstrates the retention characteristics of the 5‐bit ISPVA states, where 32 distinct current levels were stably maintained for 1000 s at −0.1 V, confirming the device's multi‐level memory capability. In addition to the pulse‐based ISPVA operation, Figure S20a,b illustrates DC‐mode multi‐level characteristics achieved by controlling the CC during the set process [71, 72, 73, 74]. Eight distinct LRS were obtained by varying CC values from 100 to 450 µA, confirming precise current‐level modulation in the nonvolatile regime. The corresponding retention characteristics, measured for 8000 s, exhibited stable and well‐separated current states across all CC conditions. These results demonstrate that both pulse‐driven and DC‐driven operations provide reliable multi‐level storage capability in the ALD‐IGZO‐based VRRAM device. These nonvolatile characteristics validate the suitability of the ALD‐IGZO‐based VRRAM as a readout‐layer element in a fully hardware RC architecture. In such systems, volatile reservoirs perform nonlinear temporal processing, while the readout layer must store and linearly combine weighted outputs. The precise, linear, and reproducible multi‐level modulation achieved in the 1F Pt/ALD‐IGZO/TiN VRRAM cell provides the stable synaptic weight control required for accurate output mapping, enabling complete fully hardware‐oriented RC operation within the vertically stacked VRRAM structure. The precise multilevel control and stability provide the device's underpinnings for synaptic learning rules, which we verify via EPSC‐based plasticity and spike‐timing‐dependent plasticity (STDP). Following the nonvolatile memory operation analysis, the synaptic characteristics of the 1F Pt/ALD‐IGZO/TiN VRRAM cell were investigated under various pulse conditions to emulate biological synaptic functions. Figure S21 presents the synaptic behaviors of the 1F Pt/ALD‐IGZO/TiN VRRAM cell in the nonvolatile regime, illustrating EPSC responses under different stimulation conditions [75, 76]. As shown in Figure S21a, spike amplitude‐dependent plasticity (SADP) behavior was evaluated by applying pulse amplitudes of 4.0, 4.2, 4.4, and 4.6 V. The current increased progressively with higher spike amplitudes, indicating enhanced synaptic weight modulation. Correspondingly, Figure S21b shows the EPSC gain (A_n_/A_1_) as a function of spike amplitude, which also increased monotonically with voltage amplitude. Similarly, the spike width‐dependent plasticity (SWDP) was investigated by varying the pulse width from 0.4 to 1.0 s while maintaining a fixed amplitude. As presented in Figure S21c,d, both the EPSC intensity and corresponding gain increased with longer pulse widths, demonstrating that extended stimulation duration facilitates stronger synaptic excitation. Furthermore, spike number‐dependent plasticity (SNDP) was examined by increasing the number of applied spikes to 5, 10, 20, 40, and 60, as shown in Figure S21e,f. The EPSC response and SNDP ratio (A_n_/A_1_) exhibited a clear cumulative enhancement with increasing spike count, reflecting the gradual reinforcement of conductive filaments under repeated electrical stimulation. This behavior mimics biological Hebbian learning, in which repeated presynaptic activity strengthens synaptic connections. Collectively, these results confirm that the Pt/ALD‐IGZO/TiN VRRAM cell successfully emulates multiple forms of nonvolatile synaptic plasticity, including amplitude‐, width‐, and number‐dependent modulation of EPSC, highlighting its potential applicability in neuromorphic computing and artificial synapse systems. Figure S22 presents the STDP characteristics of the Pt/ALD‐IGZO/TiN VRRAM device, demonstrating associative learning behavior analogous to that of biological synapses [77]. As illustrated in Figure S22a, human neural structures consist of interconnected presynaptic and postsynaptic terminals that exchange spikes through synaptic junctions. This biological mechanism was emulated in the VRRAM device by applying pre‐spike pulses to the top electrode and post‐spike pulses to the bottom electrode, corresponding to the presynaptic and postsynaptic membranes, respectively. For the STDP measurement, identical pre‐/post‐spike pulse trains of −2, −1.8, −1.6, −1.4, −1.2, and −1 V were applied with a width and interval of 100 µs, as shown in Figure S22b,c. The time difference between the pre‐ and post‐spikes (Δt = t_pre_ − t_post_) was varied to evaluate timing‐dependent conductance modulation, and the change in synaptic weight (ΔW) was calculated using Equation (6):

(6) $$\Delta W=\frac{G_{f}-G_{i}}{G_{i}}\times 100\left(\%\right)$$

Here, G_f_ and G_i_ represent the initial and final conductance values before and after pulse application, respectively. The conductance G obtained from the input pulse trains was substituted into Equation (6) to evaluate the corresponding ΔW. As shown in Figure S22d, ΔW increased as Δt decreased, indicating that the conductance modulation became stronger with closer temporal correlation between the pre‐ and post‐spikes. Specifically, long‐term potentiation (LTP) occurred when Δt > 0 (the pre‐spike was applied before the post‐spike), whereas long‐term depression (LTD) was observed when Δt < 0 (the post‐spike preceded the pre‐spike). This result implies that the stronger temporal correlation between the two spikes enhances the modulation of conductive filaments, leading to reinforced synaptic coupling. Such Δt‐dependent learning behavior is consistent with Hebbian learning theory, confirming that the Pt/ALD‐IGZO/TiN VRRAM device successfully reproduces time‐correlated learning and memory functionalities.

### 2.4 Hardware‐Oriented Wide RC System and Pattern Recognition Performance

**FIGURE 7:**
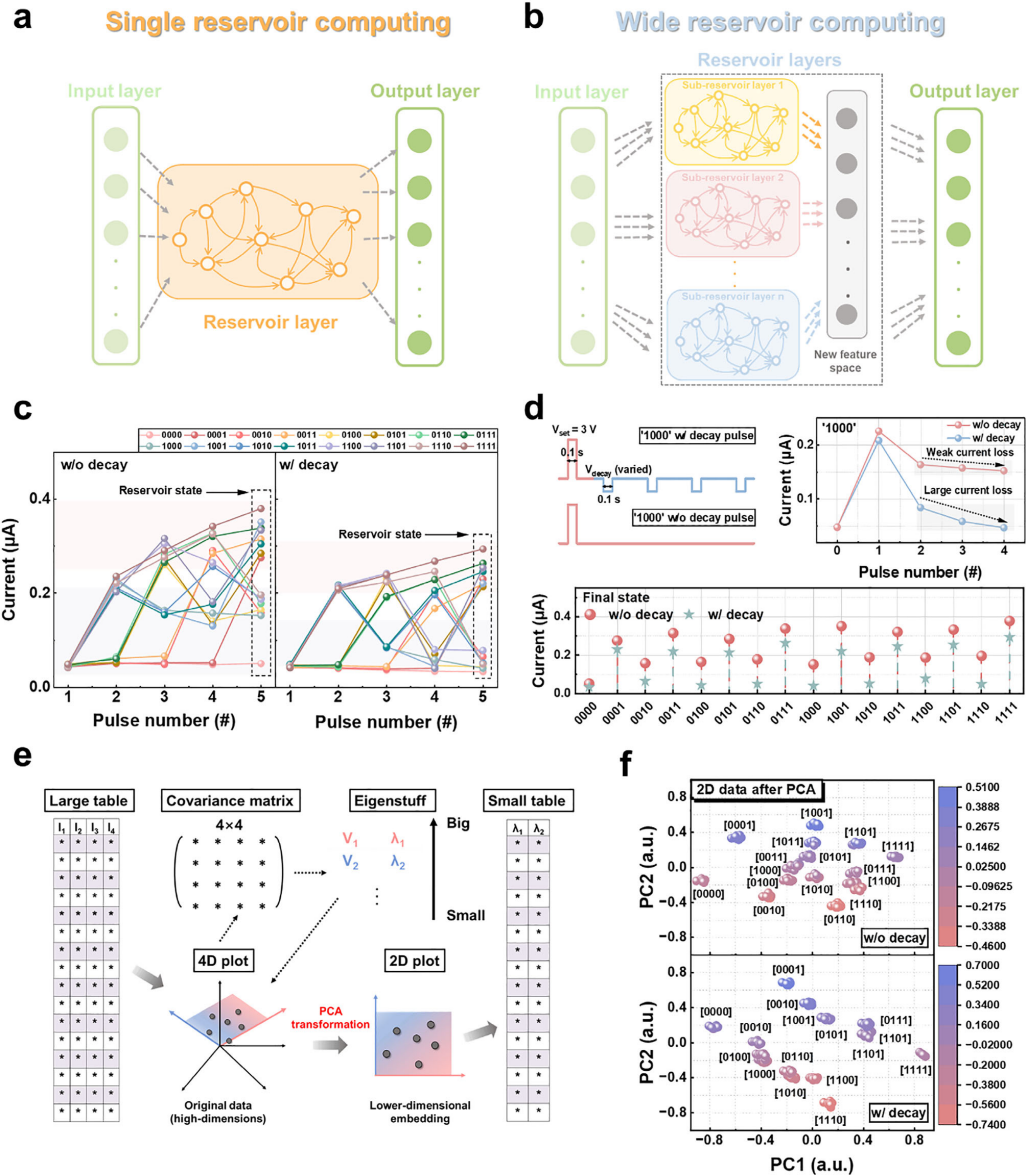
a) Schematic illustration of the single RC system, in which a single reservoir layer processes input signals with a fixed nonlinearity. b) Conceptual schematic of the proposed wide RC system, integrating multiple sub‐reservoir layers for parallel feature extraction under different decay conditions. c) Current responses of 16 states corresponding to 4‐bit input pulse streams ranging from [0000] to [1111], measured with and without decay pulses. d) Enlarged current response for the “1000” input and final current states for all 16 input combinations. e) Schematic illustration of the PCA process. f) 2D PCA projection of 4D current response vectors measured under conditions with and without decay pulses.

Having established device‐level plasticity, we next map these behaviors onto the system‐level RC framework and extend the concept to a wide RC configuration composed of parallel sub‐reservoirs exhibiting distinct decay responses. RC is a neuromorphic computing paradigm that leverages the nonlinear dynamic behavior of physical devices to process information efficiently. An RC system typically consists of an input layer, a reservoir layer, and a readout layer. The input layer encodes temporal input signals into the reservoir, where they are nonlinearly transformed into a high‐dimensional feature space. The readout layer then linearly combines these reservoir states to generate the output. Because only the readout layer weights are trained using linear regression, RC achieves highly efficient computation with minimal training overhead while maintaining strong nonlinear processing capability. Figure 7a,b compare the conceptual structures of a single RC and a wide RC system. In the single RC, as shown in Figure 7a, input data are processed through a single reservoir node exhibiting one fixed nonlinearity, which limits the diversity of extracted features [78, 79]. In contrast, the wide RC architecture, as illustrated in Figure 7b, incorporates multiple sub‐reservoir layers operating in parallel under distinct volatility conditions, enabling parallel feature extraction under varying decay conditions [26]. This parallel operation broadens the internal feature space and enhances the mapping diversity of the system, thereby compensating for the limited computational capacity of a single reservoir. To experimentally realize this concept, the Pt/ALD‐IGZO/TiN VRRAM cell before forming was employed as a physical reservoir. Before forming, conductive filaments are not generated, and transient current decay arises from local charge trapping/detrapping and carrier relaxation within the IGZO layer. This volatile behavior, in which the current gradually decreases after pulse removal, mimics the short‐term characteristics observed in biological synapses. A pulse scheme incorporating decay pulses was applied to modulate the short‐term dynamic response of the device. Figure 7c,d summarize the 4‐bit RC responses corresponding to input pulse streams ranging from [0000] to [1111], measured under conditions with and without decay pulses. Figure 7c presents all 16 states measured under both conditions. The detailed current responses corresponding to the 4‐bit input pulse streams ranging from [0000] to [1111] are provided in Figure S23 for measurements performed without decay pulses, and in Figure S24 for measurements performed with decay pulses. When decay pulses were applied, the overall current levels decreased due to faster conductance relaxation. In both cases, however, each input state exhibited distinct and well‐separated current levels, confirming stable and reproducible multistate behavior. Figure 7d magnifies the response of the representative input “1000” from Figure 7c, highlighting the difference in current decay between the two cases. With decay pulses, the current rapidly decreased after each input pulse, showing stronger volatility and greater current loss, whereas the no‐decay case exhibited slower relaxation. This behavior confirms that the decay pulse enables precise control of the memory retention time and fading strength of the reservoir. The same panel also compares the final current states of all 16 input combinations under both conditions, confirming that the reservoir's fading strength can be systematically tuned by external pulse schemes, thereby enriching short‐term dynamics and improving nonlinear feature mapping within the wide RC architecture. To evaluate the ability of the device to retain input history and to distinguish between different input patterns, Principal Component Analysis (PCA) was independently performed on reservoir states obtained under two operating conditions: without decay pulses (w/o decay) and with decay pulses (w/ decay). Each reservoir state was defined as a 4D vector, in which each dimension corresponds to the bit‐wise current response induced by a 4‐bit stimulus sequence. As schematically illustrated in Figure 7e, PCA was carried out by first constructing a covariance matrix from the 4D state vectors, followed by eigen‐decomposition to obtain the eigenvalues and their corresponding eigenvectors. The original high‐dimensional data were then projected onto a 2D subspace defined by the two principal components associated with the largest eigenvalues. The resulting 2D PCA projection is shown in Figure 7f. The first principal component (PC1) represents the overall magnitude of accumulated stimuli, primarily reflecting the strength of conductance potentiation determined by the frequency of pulses corresponding to the logical state “1”. In contrast, the second principal component (PC2) captures decay‐related temporal dynamics, arising from the interplay between information loss due to conductance relaxation and newly applied input pulses. Consequently, the reservoir states are distributed along the PC1 axis according to the number of activated bits, while input patterns with an identical pulse count are further dispersed along the PC2 axis. This behavior indicates that stimulus‐induced potentiation and decay‐driven temporal dynamics constitute orthogonal sources of variance in the 4‐bit reservoir states, highlighting the role of decay‐controlled volatility in expanding the reservoir state space and enhancing separability in the proposed wide RC architecture. Overall, the ALD‐IGZO‐based VRRAM cell demonstrated that distinct short‐term dynamic responses can be realized simply through external pulse control, enabling parallel feature extraction under different decay conditions directly in hardware. Furthermore, the proposed fully hardware‐oriented wide RC system integrates both volatile and nonvolatile functionalities within a single vertically stacked device platform: the reservoir layer utilizes the volatility of cells before forming for short‐term dynamics, while the readout layer employs ISPVA‐based 6‐bit programmable conductance states to store trained weights through nonvolatile switching. This integration enables hardware‐implemented inference and in‐memory storage of the trained readout weights, thereby supporting energy‐efficient and stable RC operation.

**FIGURE 8:**
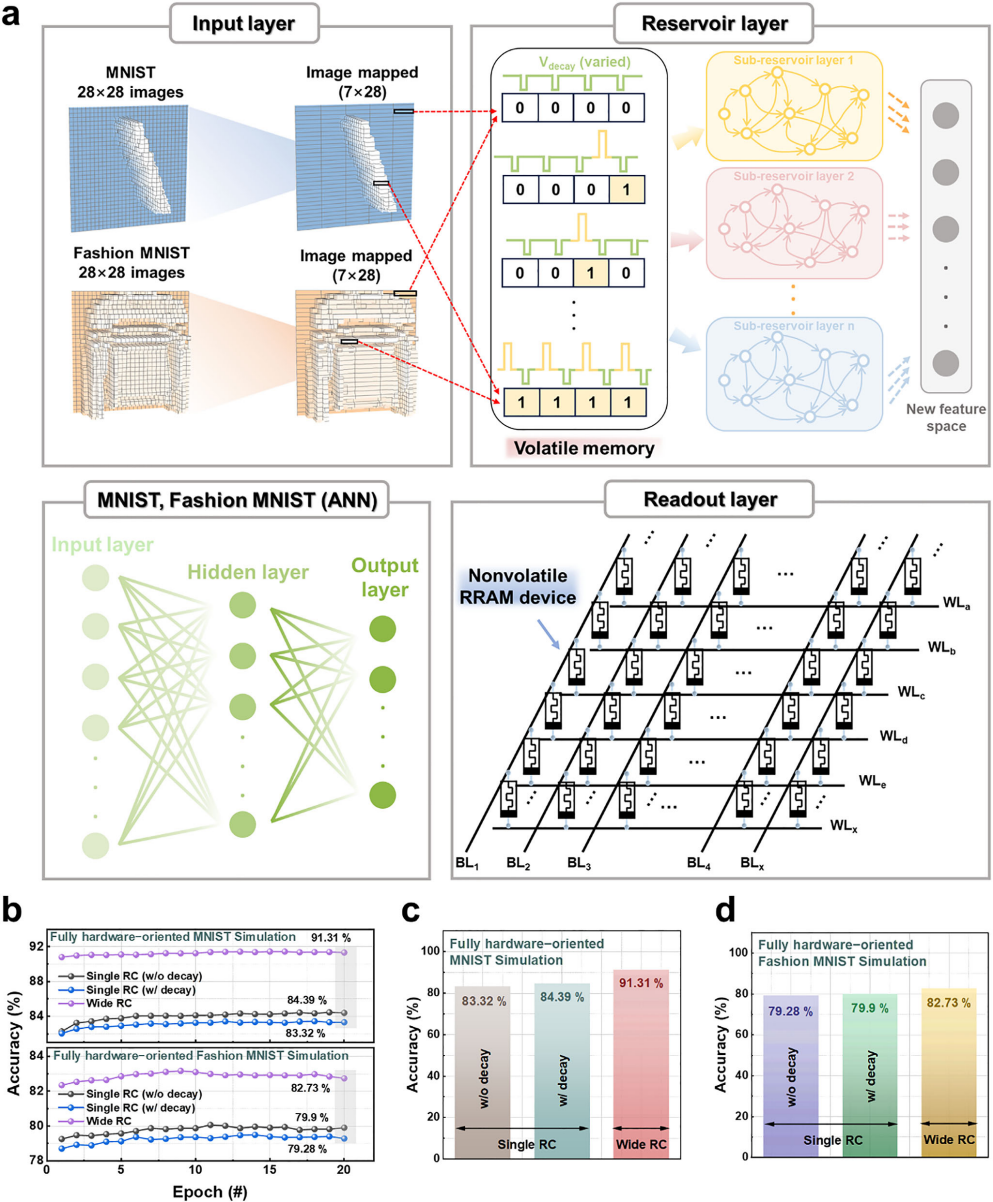
a) Schematic illustration of the fully hardware‐oriented wide RC system based on ALD‐IGZO VRRAM, comprising an input layer, a reservoir layer, and a readout layer. b) Comparison of recognition accuracies between the single RC and wide RC systems for the MNIST and Fashion MNIST datasets. c) Bar chart of the MNIST simulation results for the hardware‐oriented wide RC system. d) Bar chart of the Fashion MNIST simulation results for the hardware‐oriented wide RC system.

Figure 8 presents the overall architecture and operational characteristics of the fully hardware‐oriented wide RC system based on ALD‐IGZO VRRAM. The system employs volatile VRRAM cells maintained in the unformed state as the reservoir layer and physically distinct formed VRRAM cells exhibiting nonvolatile characteristics as the readout layer. Through this configuration, both STM and LTM functionalities are realized within the same vertically stacked ALD‐IGZO VRRAM platform. Figure 8a illustrates the input and reservoir operation. MNIST and Fashion‐MNIST images (28 × 28 pixels) were divided into 7 × 28 subgroups and converted into 4‐bit pulse streams ([0000]–[1111]) [80, 81, 82]. These sequences were applied to the volatile Pt/ALD‐IGZO/TiN VRRAM cells before forming, where each pulse train induced a distinct current‐decay response depending on the applied decay‐bias condition. The resulting 16 current‐response states served as physical reservoir nodes distributed across multiple sub‐reservoir layers operating in parallel, forming a high‐dimensional feature space [83]. Each sub‐reservoir exhibited unique decay‐dependent transient behavior, enabling mapping of input patterns into diverse short‐term dynamic features. The readout layer employed nonvolatile VRRAM cells after forming to implement stable LTM and preserve the trained weights. During the training phase, the transient current responses generated by the unformed volatile VRRAM cells were concatenated to construct the reservoir state vector (*x*), which was subsequently used for offline ANN/MLP‐based readout training. The resulting weight matrix (*W*) was mapped onto and physically programmed into the multilevel conductance states of the formed nonvolatile VRRAM cells. During inference, an unseen input sequence generated a new reservoir state vector, and the measured reservoir responses were converted into the corresponding voltage levels and simultaneously applied to the individual top electrodes of the programmed nonvolatile readout cells. The resulting weighted currents were physically accumulated at the shared bottom electrode according to Kirchhoff's current law, thereby implementing the reservoir‐to‐readout multiply–accumulate operation in the VRRAM network rather than through numerical software calculation. Python was used for offline readout training, instrument control, reservoir‐response scaling, conductance programming, data acquisition, and output‐label assignment, whereas the central multiply–accumulate operation was physically performed by the programmed VRRAM network. The overall training and hardware‐implemented inference workflow is illustrated in Figure S25, and the corresponding system‐level operation and experimental hardware interconnection are described in Notes S2 and S3, respectively. Figure 8b presents the simulation results, revealing that the proposed wide RC structure achieved significantly higher recognition accuracy than the single RC configuration. For the MNIST dataset, the wide RC achieved 91.31% accuracy, compared with 84.39% for the single RC, while for the Fashion‐MNIST dataset, the wide and single RC systems achieved 82.73% and 79.9%, respectively. The superior performance of the wide RC is attributed to the parallel operation of multiple sub‐reservoir layers with distinct decay responses, which enhances nonlinear mapping capability and state separability. Both systems exhibited higher accuracy on MNIST than on Fashion‐MNIST, as the latter contains more complex spatial features that make classification inherently more challenging [84]. Figure 8c,d show bar charts comparing MNIST and Fashion‐MNIST recognition results, further confirming that parallel operation of sub‐reservoir layers enhances classification performance. The confusion‐matrix analyses presented in Figure S26a,b further verify stable and well‐distributed classification performance for the MNIST and Fashion‐MNIST datasets, respectively, across all categories. To quantitatively evaluate the energy efficiency of the proposed hardware RC system, the energy consumption was estimated using the same pulse conditions employed in the reservoir computing experiments. Based on an input pulse amplitude of 2 V, a pulse width of 100 µs, and an experimentally measured average current of 94.456 nA, the energy consumption of a single reservoir input pulse was estimated to be 18.89 pJ. Since each MNIST image is processed using 196 sequential input pulses, the device‐level energy consumed during the reservoir operation was estimated to be 3.70 nJ per classification. A comparison with representative hardware reservoir computing systems is summarized in Table S1, while the detailed calculation procedure is provided in Note S1. These findings demonstrate that short‐term dynamic features extracted under varying decay conditions can be effectively encoded at the hardware level. Consequently, the proposed wide RC system, which leverages the volatile and nonvolatile characteristics of ALD‐IGZO VRRAM, represents a promising fully hardware‐oriented neuromorphic computing platform capable of achieving low‐power operation and high recognition accuracy. Table 1 summarizes the key characteristics of previously reported RC systems and the proposed ALD‐IGZO‐based vertical wide RC structure. Unlike conventional RC platforms that rely on planar or partially software‐assisted configurations, the proposed system physically implements the volatile reservoir transformation, nonvolatile weight storage, and reservoir‐to‐readout multiply‐accumulate operation using a vertically stacked VRRAM architecture. The use of ALD ensures uniform and conformal coating of IGZO along the sidewall surfaces of the vertical contact holes, guaranteeing excellent film uniformity and reproducibility within the stacked VRRAM structure. Furthermore, by employing a wide RC architecture composed of multiple sub‐reservoir layers operating under different volatility conditions, the system achieves enhanced nonlinear mapping and feature‐separation capability compared with single‐layer RC systems. This parallel operation of sub‐reservoirs effectively expands the computational dimensionality while maintaining low power consumption and a compact structural design. In addition, the vertical stacking configuration enables the integration of volatile and nonvolatile functionalities within a single 2F VRRAM device, allowing short‐term and long‐term memory behaviors to coexist in a fully hardware‐oriented neuromorphic platform [85, 86, 87]. Consequently, the proposed ALD‐IGZO VRRAM‐based wide RC system offers significant advantages in scalability, fabrication compatibility, and energy efficiency, providing a promising foundation for next‐generation hardware neuromorphic computing.

**TABLE 1:** Comparison of deposition methods for IGZO‐based devices and their RC performance.

Devices	Stacking	Method	RC architecture	Application	Accuracy	Ref.
Al/IGZO/ZrO*_x_*/Pt	No	PVD	Single (in‐sensor RC)	Digit recognition	No	[88]
Al/Al_2_O_3_/ZnO/Al_2_O_3_/IGZO/Al	No	PVD	Single (temporal adaptive RC)	Human activity recognition	84.2%−96.7%	[22]
ITO/NiO/IGZO/Ti/Pt	No	PVD	Single (static RC) Single (dynamic RC)	MNIST classification Spoken‐digit recognition	95.2% 98.2%	[89]
Pt/IGZO/SnO_x_/TiN	No	PVD	Single (static RC)	Digit recognition	>95%	[90]
Mo/IGZO/HZO/n^+^Si	No	PVD	Single (static RC)	MNIST classification	90.14%	[24]
Ti/IGZO/TaO_x_/SiO_2_/p^+^Si	No	PVD	Single (static RC) Single (static RC)	MNIST classification Fashion MNIST classification	95.75% 85.02%	[91]
ITO/IGZO/TaN	No	PVD	Single (static RC)	MNIST classification	94.1%	[92]
ITO/ZnO/IGZO/ZnO/ITO	No	PVD	Single (static RC)	MNIST classification	78.79%	[93]
Mo/IGZO/Mo	No	PVD	No	No	No	[94]
MoW/a‐IGZO/TiO_2_/n‐type Si	No	PVD	No	No	No	[95]
Cu/TiN/IGZO/ZnO/ITO	No	PVD	No	No	No	[96]
W/IGZO/HfAlO_x_/CeO_x_/W	No	PVD	No	No	No	[97]
Pt/ALD‐IGZO/TiN	Yes	ALD	Wide (dynamic RC)	MNIST classification Fashion MNIST classification	91.31% 82.73%	This work

## 3 Conclusion

In this work, we demonstrated a fully hardware‐oriented wide RC system based on a vertically integrated ALD‐IGZO VRRAM structure. Owing to the conformal step coverage and atomic‐level compositional controllability of ALD, the IGZO switching layer was uniformly formed along the vertical sidewall, enabling the reliable realization of both volatile and nonvolatile conduction states within a single device. These material and process advantages ensured highly reproducible device characteristics, which are essential for scalable neuromorphic hardware. The volatile state before forming served as the reservoir layer, exhibiting short‐term fading memory dynamics governed by charge trapping and detrapping, whereas the nonvolatile state after forming functioned as the readout layer, providing controllable long‐term weight modulation through the ISPVA method. By leveraging multiple sub‐reservoir layers with distinct volatility, the proposed wide RC architecture achieved enhanced nonlinear mapping and feature separation, resulting in high pattern recognition accuracy of 91.3% on the MNIST dataset with an estimated energy consumption of 3.70 nJ per classification. Furthermore, the ALD‐grown IGZO demonstrated superior thin‐film uniformity and stable switching behavior across the 3D stacked structure, validating the suitability of ALD‐based oxide semiconductors for vertically integrated neuromorphic systems. Overall, this study establishes a scalable and energy‐efficient in‐memory computing framework that unifies short‐ and long‐term dynamics within a single ALD‐IGZO VRRAM platform, paving the way toward practical, adaptive, and learning‐capable hardware reservoir systems.

## 4 Experimental Section

The Pt/ALD‐IGZO/TiN stacked VRRAM device was fabricated following the process sequence illustrated in Figure S1. First, a 4‐inch p‐type silicon wafer with a 300 nm‐thick SiO_2_ layer grown by wet oxidation was prepared. Surface organic residues and metallic contaminants were removed using the standard SPM cleaning process. Subsequently, the TiN and SiO_2_ layers were alternately deposited twice to form the 1F and 2F structures. The TiN planar electrode was deposited to a thickness of 100 nm by reactive sputtering (SRN120), and the SiO_2_ insulating layer was deposited using PECVD (PLASMAPRO SYSTEM100). Cylindrical holes with a diameter of 10 µm were then patterned by photolithography and reactive ion etching (RIE, Oxford RIE 80 Plus). After etching, a PR Asher (Plasma Finish, V15‐G) was used to remove any residual etching byproducts. Next, a 5 nm‐thick IGZO film was uniformly deposited over the entire surface via ALD. The IGZO thin films were deposited using PEALD. Indium, gallium, and zinc oxide sub‐layers were formed using DADI, TMG, and DEZ as the respective metal precursors. The deposition was carried out at a stage temperature of 150°C and a chamber pressure of 10 mTorr. Each metal‐oxide sub‐cycle consisted of precursor dosing, purge, oxygen stabilization, and plasma exposure steps. Specifically, the InO_x_ sub‐cycle comprised a 0.5 s DADI dose, 20 s purge, 5 s O_2_ stabilization, and 1.5 s plasma exposure. Due to the high delivery rate of the DADI precursor, a small‐aperture gasket was used to regulate precursor injection. The GaO_x_ sub‐cycle included a 0.5 s TMG dose, 5 s purge, 5 s O_2_ stabilization, and 2 s plasma exposure, while the ZnO_x_ sub‐cycle consisted of a 0.5 s DEZ dose, 10 s purge, 5 s O_2_ stabilization, and 4.5 s plasma exposure. These sub‐cycles were sequentially repeated according to the designated super‐cycle to form the complete IGZO layer. Photolithography and etching processes (Oxford etch) were repeated twice to expose the 1F and 2F metal layers. Finally, after photolithography for pillar electrode patterning, a 100 nm‐thick Pt layer was deposited using an E‐beam evaporator, followed by a lift‐off process to complete the fabrication of the Pt/ALD‐IGZO/TiN VRRAM device. The electrical characteristics of the fabricated devices were evaluated using a Keithley 4200‐SCS semiconductor parameter analyzer (SPA) and a 4225‐PMU ultrafast module (Solon, OH, USA) in a probe station. During electrical measurements, the planar electrodes of each layer were grounded, while the programming, erasing, and reading voltages were applied to the pillar electrode. Unless otherwise stated, electrical measurements were conducted at room temperature and ambient pressure. Both DC sweep and transient analyses were performed. The cross‐sectional structure of the fabricated devices was examined using TEM, and the elemental composition was analyzed by EDS and XPS.

## Author Contributions

**Seohyeon Ju**: investigation, data curation, formal analysis. **Seong‐Cheol Jang**: investigation, formal analysis, data curation. **Nawoon Kim**: investigation, formal analysis, data curation. **Hyun‐Suk Kim**: writing – review and editing, project administration, funding acquisition, conceptualization, supervision. **Jaewon Park**: investigation, formal analysis, visualization, writing – original draft. **Sungjun Kim**: conceptualization, project administration, supervision, funding acquisition, writing – review and editing. **Seeun Lee**: writing – original draft, visualization, formal analysis, investigation.

## Conflicts of Interest

The authors declare no conflicts of interest.

## Supporting information




**Supporting File**: advs77076‐sup‐0001‐SuppMat.docx.

## Data Availability

The data that support the findings of this study are available from the corresponding author upon reasonable request.
