# Supplementary material for: Coexisting Volatile and Nonvolatile Switching in 3D ALD‐IGZO Vertical RRAM for Fully Hardware‐Based Wide Reservoir Computing
Source: Adv Sci (Weinh). 2026 Aug 11;13(61):e77076. doi: 10.1002/advs.77076 (PMC13460410; doi:10.1002/advs.77076)
Supplement: Supplementary file 1 [file ADVS-13-e77076-s001.docx]

Supporting Information

**Coexisting Volatile and Nonvolatile Switching in 3D ALD-IGZO Vertical RRAM for Fully Hardware-Based Wide Reservoir Computing**

Seeun Lee^1†^, Jaewon Park^2†^, Nawoon Kim^1^, Seohyeon Ju^1^, Seong-Cheol Jang^2^, Hyun-Suk Kim^2*^, Sungjun Kim^1*^

^1^Division of Electronics and Electrical Engineering, Dongguk University, Seoul 04620, Republic of Korea

^2^Department of Energy and Materials Engineering, Dongguk University, Seoul 04620, Republic of Korea

E-mail: [sungjun@dongguk.edu](mailto:sungjun@dongguk.edu), [khs3297@dongguk.edu](mailto:khs3297@dongguk.edu)

**Table of contents**

- **Figure S1-S26**
- **Table S1**
- **Supplementary Note 1-3**
- **Supplementary References**

**Supplementary Figures**

**Figure S1.** Fabrication process of Pt/ALD-IGZO/TiN VRRAM device.

**Figure S2.** a) Single-cell device structure and experimental matrix of IGZO films with varying thickness and In:Ga:Zn composition. b) Metallic-like I–V characteristics of devices 1-10. c) Stable resistive switching behavior of Ga-rich IGZO devices (devices 11 and 12, In:Ga:Zn = 1:3:1).

**Figure S3.** a) Cross-sectional SEM image showing the hole diameter of the VRRAM structure. b) Cross-sectional SEM image illustrating the hole slope. c) Cross-sectional schematic depicting the hole diameter and sidewall angle in the VRRAM structure.

**Figure S4.** Extended volatile switching endurance characteristics of the 1F VRRAM cell. a) Magnified views of the initial (cycles 1-100), intermediate (cycles 24 951-25 050), and final (cycles 49 901-50 000) regions of the 50 000-cycle endurance test. b) Conductance modulation window (ΔG) as a function of cycle number. Each data point represents the mean ΔG over 100 consecutive cycles, and the dashed line denotes the overall mean value of 0.222 μS.

**Figure S5.** Electrical characteristics of the 2F Pt/ALD-IGZO/TiN VRRAM cell exhibiting volatile behavior before forming process. a) Consecutive I–V curves measured over 10 sweep cycles. b) Pulse endurance characteristics of the volatile switching behavior measured over 50 000 consecutive cycles at a read voltage of 2 V. c) Decay behavior of the on-state current monitored at a read voltage of −1 V for 10 000 s. d) P&D characteristics under consecutive pulse stimulation.

**Figure S6.** Cell-to-cell volatile I–V characteristics measured from ten different devices before the forming process. a) 1F layer. b) 2F layer.

 **Figure S7**. Statistical comparison of the electrical characteristics between the 1F and 2F VRRAM cells before the forming process. a) Cell-to-cell distributions of the HRS and LRS resistances measured from ten different devices in the 1F and 2F layers under a read voltage of 2 V. b) Cumulative probability distributions of the HRS and LRS resistances.

**Figure S8.** a) Schematic of the pulse scheme used to observe P&D. b) P&D10-cycle repeatable behavior of the Pt/ALD-IGZO/TiN VRRAM device acquired through the demonstrated pulse scheme.

**Figure S9.** a) Schematic of the pulse scheme used P&D under different pulse number conditions. b) P&D behavior measured under different pulse numbers. c) Schematic of the pulse scheme used for P&D with different pulse widths. d) P&D behavior measured under different pulse widths.

**Figure S10.** Memory decay behavior and variation of decay constant under different V_bias_ of a) −1 V, b) −0.5 V, c) 0 V, d) 0.5 V, e) 1 V.

**Figure S11.** Cycle-to-cycle uniformity of the volatile characteristics measured over 50 consecutive cycles at V_bias_ = −1 V. a) Overlay of the transient current decay responses measured over 50 consecutive cycles, demonstrating reproducible volatile relaxation behavior. b) Extracted decay time constant ($\tau$) as a function of cycle number, showing stable fading-memory characteristics. c) Cycle-to-cycle variation of the transient response amplitude ($\Delta I=I_{peak}-I_{baseline}$), indicating highly reproducible volatile responses.

**Figure S12.** UPS spectra and work-function determination of a) Pt, b) TiN, c) Pt/IGZO, and d) TiN/IGZO.

**Figure S13**. a) Temperature-dependent I–V characteristics of the Pt/ALD-IGZO/TiN VRRAM measured from 25 to 125 ºC. b) Arrhenius plot showing ln(I/T^2^) versus 1/T curves for the HRS, LRS states measured at the same read voltage of 2 V.

**Figure S14.** Schematic of the switching process of a) initial state, b) forming, c) set and d) reset in the Pt/ALD-IGZO/TiN VRRAM device.

**Figure S15.** Electrical characteristics of the 2F Pt/ALD-IGZO/TiN VRRAM cell exhibiting nonvolatile behavior after the forming process. a) Consecutive I–V curves measured over 10 sweep cycles including the forming process. b) Endurance characteristics measured under a read voltage of −0.1 V. c) Retention characteristics obtained at a read voltage of −0.1 V for 10 000 s. d) CV values of the HRS and LRS obtained from consistent DC switching cycles.

**Figure S16.** Cell-to-cell nonvolatile I–V characteristics measured from ten different devices after the forming process. a) 1F layer. b) 2F layer.

**Figure S17**. Statistical comparison of the electrical characteristics between the 1F and 2F VRRAM cells after the forming process. a) Cell-to-cell distributions of the HRS and LRS resistances measured from ten different devices in the 1F and 2F layers under a read voltage of −0.1 V. b) Cumulative probability distributions of the HRS and LRS resistances.

**Figure S18**. Retention characteristics of the 1F Pt/ALD-IGZO/TiN VRRAM cell measured at R.T., 85 °C, and 125 °C for 10 000 s at a read voltage of −0.1 V.

**Figure S19.** Statistical evaluation of 6-bit conductance modulation characteristics using ISPVA. a) 6-bit potentiation and depression tuning characteristics measured from 10 independently fabricated VRRAM cells. b,c) Adjacent-state margin (ΔG/σ_avg_) analysis for potentiation and depression states, respectively. d,e) Mean conductance and standard deviation extracted from ten VRRAM cells during potentiation and depression, respectively. The average CV values were 1.02% and 1.09% for potentiation and depression, respectively, and no adjacent-state overlap was observed throughout the entire 6-bit conductance range.

**Figure S20.** a) I–V curves for different values of CC in the NVM device. b) Multi-level cell retention performance measured under different values of CC.

**Figure S21.** Synaptic plasticity characteristics of the Pt/ALD-IGZO/TiN VRRAM device. a) SADP. b) EPSC gain (A_n_/A_1_) as a function of increasing spike amplitude. c) SWDP. d) EPSC gain (A_n_/A_1_) as a function of increasing spike width. e) SNDP. f) SNDP ratio (A_n_/A_1_) as a function of the number of spikes.

**Figure S22.** a) Schematic illustration of a human synaptic neural structure. b) Pulse configuration for STDP measurement at Δt = 100 μs. c) Schematic of the applied pre-/post-spike pulse sequence for STDP measurement. d) Experimental result of STDP characteristics.

**Figure S23.** Current responses of 16 states in 4-bit RC corresponding to input pulse streams ranging from [0000] to [1111], obtained without a decay pulse applied.

**Figure S24.** Current responses of 16 states in 4-bit RC corresponding to input pulse streams ranging from [0000] to [1111], obtained with a decay pulse applied.

**Figure S25.** System-level schematic of the training and hardware-implemented inference processes using the volatile reservoir and nonvolatile readout layers.

**Figure S26.** a) Confusion matrix derived from inference results of wide RC MNIST. b) Confusion matrix derived from inference results of the wide RC Fashion MNIST.

**Supplementary Table**

**Table S1.** Comparison of energy consumption with previously reported hardware reservoir computing systems.

| Devices | Application | Energy metric | Energy | Accuracy | Ref. |
| --- | --- | --- | --- | --- | --- |
| Ti/TiO_x_/TaO_y_/Pt | Spoken-digit recognition | per input | 6 nJ | 99.6% | [S1] |
| W/HZO/Si | Sequence classification | per input | 35 pJ | 92.3% | [S2] |
| Ag/HfO_2_/Pt | PCB defect classification | per classification | 16-63 nJ | 86.3% | [S3] |
| Pd/Au/WO_x_/W | MNIST digit recognition | per input | 3 nJ | 88.1% | [S4] |
| Ag nanowire network+TaO_x_ ReRAM | MNIST digit classification | per input | 750 μJ | 90.4% | [S5] |
| CMOS eRNR+memristor array | Handwritten vowel recognition | per classification | 463.36 pJ | 94.0±0.8% | [S6] |
| Au/sol-gel silica/HZO/Si | ECG arrhythmia classification | per reservoir state | 27 fJ | 97.8% | [S7] |
| Pt/ALD-IGZO/TiN | MNIST classification | per classification | 3.70 nJ | 91.3% | This work |

**Supplementary Notes**

**Supplementary Note 1.** Estimation of energy consumption

To quantitatively evaluate the energy efficiency of the proposed ALD-IGZO VRRAM-based wide reservoir computing (RC) system, the energy consumption was estimated using the same pulsed operating conditions employed in the reservoir computing experiments.

The energy consumed by a single input pulse can be expressed as

$$E_{pulse}=\int_{0}^{t_{pulse}} V\left( t \right)I\left( t \right)dt$$

where $V\left( t \right)$ and $I\left( t \right)$ are the instantaneous voltage and current during the pulse, respectively. Assuming a rectangular voltage pulse and approximating the current by the experimentally measured average current during the pulse, the pulse energy can be written as

$E_{pulse}\approx V_{pulse}\times I_{avg}\times t_{pulse}$

where $V_{pulse}$, $I_{avg}$, and $t_{pulse}$ denote the pulse amplitude, average current during the pulse, and pulse width, respectively.

For the volatile reservoir operation, the 2F ALD-IGZO VRRAM device was driven by input voltage pulses with an amplitude of 2 V and a pulse width of 100 μs. The experimentally measured average current during the pulse was 94.456 nA. Therefore, the energy consumed by a single reservoir input pulse can be estimated as

$$E_{pulse}=2 V\times94.46 nA\times100 \mu s=1.889\times{10}^{-11} J=18.89 pJ$$

In the proposed hardware RC architecture, each MNIST image is converted into 196 sequential input pulses through the 4-bit input encoding scheme. Accordingly, the total energy consumption per image classification is given by

$$E_{class}=N_{pulse}\times E_{pulse}$$

where $N_{pulse}$ is the number of input pulses per image.

Substituting $N_{pulse}=196$,

$$E_{class}=196\times18.89 pJ=3.70\times{10}^{-9} J=3.70 nJ$$

Therefore, the proposed ALD-IGZO VRRAM-based wide RC system consumes an estimated 3.70 nJ per MNIST classification under the experimentally employed operating conditions. The estimated classification energy is summarized in Table S1 together with representative hardware reservoir computing systems, providing a quantitative benchmark for evaluating the energy efficiency of the proposed ALD-IGZO VRRAM-based wide RC architecture.

**Supplementary Note 2.** Interconnection between the volatile reservoir and the nonvolatile readout layer

Figure S25 illustrates the operational flow of the proposed hardware reservoir computing (RC) system based on the dual-mode operation of the ALD-IGZO VRRAM device. Rather than employing separate device technologies for the reservoir and readout layers, the proposed architecture utilizes physically distinct cells fabricated within the same VRRAM platform, exploiting the volatile switching behavior before forming and nonvolatile switching behavior after forming.

During the training phase, the encoded input pulse sequence is applied to the volatile VRRAM cells before forming. Owing to the intrinsic conductance relaxation originating from charge trapping/detrapping, the transient current responses generated by the reservoir are concatenated to construct the reservoir state vector ($x$). This reservoir state vector is subsequently used as the input for ANN/MLP-based readout training to determine the optimal readout weight matrix ($W$). The trained weight matrix is then mapped onto the multilevel conductance states of the nonvolatile VRRAM cells after forming using the ISPVA programming method, enabling stable long-term storage of the trained readout weights.

During the inference phase, an unseen input pulse sequence is processed by the volatile VRRAM reservoir in the same manner, generating a new reservoir state vector ($x$). The measured reservoir responses are converted into the corresponding voltage levels and applied to the individually programmed nonvolatile VRRAM readout cells. The programmed conductance states of the nonvolatile VRRAM cells serve as fixed readout weights ($W$), and the final output is obtained through the linear readout operation,

$$y=Wx$$

where $x$ represents the reservoir state vector and $W$ denotes the trained weight matrix stored in the nonvolatile VRRAM cells. During this operation, the multiplication between the reservoir-state voltages and the programmed conductance values, as well as the accumulation of the resulting weighted currents, is physically performed by the nonvolatile VRRAM readout network.

Accordingly, the volatile reservoir and the nonvolatile readout layer are functionally interconnected through the transfer of the reservoir state vector during inference and the mapping of the trained readout weights onto the nonvolatile conductance states, as schematically illustrated in Figure S25. The detailed experimental hardware connection and signal-transfer procedure are described in Supplementary Note 3. This architecture enables short-term temporal information processing and long-term weight storage to be realized within the same ALD-IGZO VRRAM platform by assigning unformed and formed VRRAM cells to the reservoir and readout functions, respectively.

**Supplementary Note 3.** Experimental hardware interconnection and signal transfer between the volatile reservoir and nonvolatile readout

The volatile reservoir and nonvolatile readout functions were experimentally implemented using physically distinct VRRAM cells fabricated on the same device platform. Cells maintained in the unformed state were used as the volatile reservoir, whereas formed cells exhibiting stable multilevel conductance states were used as the nonvolatile readout. Electrical measurements and automated signal transfer were controlled using a Keithley 4200-SCS semiconductor parameter analyzer equipped with a 4225-PMU ultrafast module. An automated Python program communicating with the measurement system through the Keithley External Control Interface (KXCI) and VISA was used to execute the pulse sequences, acquire the measured currents, convert the measured reservoir responses into readout-voltage commands, and control the subsequent readout operation.

During the reservoir operation, the encoded input pulse sequences were applied to the volatile VRRAM cells before forming, and the resulting transient current responses were experimentally measured as the reservoir states. The measured current responses were normalized and converted into the corresponding readout voltage levels according to the scaling procedure used during system training. These voltage commands were subsequently generated by the measurement system and applied to the individual top electrodes of the nonvolatile readout cells. Thus, the reservoir responses were transferred to the readout layer through an automated measurement-and-control sequence without manual spreadsheet export or software-based calculation of the readout multiply–accumulate operation.

The readout weight matrix obtained through offline ANN/MLP training was mapped onto the available conductance range of the nonvolatile VRRAM cells. Each target weight was physically stored as a programmed conductance state using the incremental step pulse with verify algorithm (ISPVA). During inference, the reservoir-state voltages were simultaneously applied to the individually programmed top electrodes. For each output-channel measurement, the corresponding set of readout cells shared a common bottom electrode that served as a physical current-summing node. The current generated through each cell was determined by the applied reservoir-state voltage and the programmed conductance state. Accordingly, the output current for channel $j$ was expressed as:

$$I_{j}=\sum_{i=1}^{N} V_{i}G_{ij}$$

where $V_{i}$ is the voltage corresponding to the $i$-th reservoir state and $G_{ij}$ is the programmed conductance representing the readout weight between reservoir node $i$ and output channel $j$. The individual weighted currents were physically accumulated at the common bottom electrode node according to Kirchhoff’s current law, and the resulting output current was directly measured using the semiconductor parameter analyzer.

Accordingly, neither the multiplication between the reservoir-state voltages and the programmed conductance values nor the summation of the individual cell currents was numerically performed in Python during inference. Python was used only for offline weight training, instrument control, pulse-sequence generation, reservoir-response scaling, conductance programming, data acquisition, and determination of the predicted class based on the directly measured hardware output currents. The central reservoir-to-readout multiply–accumulate operation was physically executed by the programmed VRRAM network rather than calculated in software.

The present proof-of-concept implementation employed external semiconductor measurement instrumentation rather than custom-designed or monolithically integrated peripheral circuits. Therefore, the term “fully hardware-based” in this work specifically refers to the physical implementation of the volatile reservoir transformation, nonvolatile weight storage, and reservoir-to-readout multiply–accumulate operation using the fabricated VRRAM devices. Offline training, measurement sequencing, reservoir-response scaling, data acquisition, and output-label assignment were performed using an external PC and measurement instrumentation. The overall training and inference workflow is illustrated in Figure S25, whereas the present note clarifies the corresponding experimental hardware connection and signal-transfer procedure.

**Supplementary References**

[S1]	Y. Zhong, J. Tang, X. Li, et al., “Dynamic memristor-based reservoir computing for high-efficiency temporal signal processing,” *Nature Communications* 12 (2021): 408, <https://doi.org/10.1038/s41467-020-20692-1>.

[S2]	J. Yu, E. Covi, S. Slesazeck, et al., “Energy efficient and robust reservoir computing system using ultrathin (3.5 nm) ferroelectric tunneling junctions for temporal data learning,” *IEEE Electron Device Letters* 42 (2021): 1080–1083, https://doi.org/10.1109/LED.2021.3082856.

[S3]	Y. Lee, W. Lee, J. Shin, et al., “Physical Reservoir Computing for Smart Manufacturing of PCB Using Crystallinity-Controlled HfO_2_ Memristor for Reservoir,” *Advanced Intelligent Systems* 8 (2026): 2501045, <https://doi.org/10.1002/aisy.202501045>.

[S4]	C. Du, F. Cai, M. A. Zidan, et al., “Reservoir computing using dynamic memristors for temporal information processing,” *Nature Communications* 8 (2017): 2204, <https://doi.org/10.1038/s41467-017-02337-y>.

[S5]	G. Milano, G. Pedretti, K. Montano, et al., “In materia reservoir computing with a fully memristive architecture based on self-organizing nanowire networks,” *Nature Materials* 21 (2022): 195–202, <https://doi.org/10.1038/s41563-021-01099-9>.

[S6]	X. Liang, Y. Zhong, J. Tang, et al., “Rotating neurons for all-analog implementation of cyclic reservoir computing,” *Nature Communications* 13 (2022): 1549, <https://doi.org/10.1038/s41467-022-29260-1>.

[S7]	S. Jiang, X. Wang, Y. Zhang, et al., “Energy-Efficient Reservoir Computing Based on Solution-Processed Electrolyte/Ferroelectric Memcapacitive Synapses for Biosignal Classification,” *The Journal of Physical Chemistry Letters* 15 (2024): 8501–8509, https://doi.org/10.1021/acs.jpclett.4c01896.
